# Evolutionary adaptations of doublet microtubules in trypanosomatid parasites

**DOI:** 10.1126/science.adr5507

**Published:** 2025-03-14

**Authors:** Matthew H. Doran, Qingwei Niu, Jianwei Zeng, Tom Beneke, James Smith, Peter Ren, Sophia Fochler, Adrian Coscia, Johanna L. Höög, Shimi Meleppattu, Polina V. Lishko, Richard J. Wheeler, Eva Gluenz, Rui Zhang, Alan Brown

**Affiliations:** 1 Department of Biological Chemistry and Molecular Pharmacology, Blavatnik Institute, Harvard Medical School, 240 Longwood Avenue, Boston, MA, USA; 2 Department of Biochemistry and Molecular Biophysics, Washington University in St. Louis School of Medicine, St. Louis, MO, USA; Molecular Cell Biology (MCB) graduate program, Division of Biology & Biomedical Sciences, Washington University in St. Louis School of Medicine, St. Louis, MO, USA; 3 Department of Biochemistry and Molecular Biophysics, Washington University in St. Louis, School of Medicine, St. Louis, MO, USA; 4 Sir William Dunn School of Pathology, University of Oxford, Oxford, OX1 3RE, UK; 7 Institute of Cell Biology, University of Bern, Baltzerstrasse 4, 3012 Bern, Switzerland; 8 Department of Chemistry & Molecular Biology, University of Gothenburg, Sweden; 9 Department of Cell Biology and Physiology, Washington University in St. Louis, School of Medicine, St. Louis, MO; 10 Medawar Building for Pathogen Research, Nuffield Department of Medicine, University of Oxford, Oxford, UK; 11 Institute for Immunology and Infection Research, School of Biological Sciences, University of Edinburgh, Ashworth laboratories, Charlotte Auerbach Road, Edinburgh, EH9 3FL, United Kingdom

## Abstract

The movement and pathogenicity of trypanosomatid species, the causative agents of trypanosomiasis and leishmaniasis, are dependent on a flagellum containing an axoneme of dynein-bound doublet microtubules (DMTs). Here, we present cryo-EM structures of DMTs from two trypanosomatid species, *Leishmania tarentolae* and *Crithidia fasciculata*, at resolutions up to 2.7 Å. The structures revealed 27 trypanosomatid-specific microtubule inner proteins, a specialized dynein-docking complex, and the presence of paralogous proteins that enable higher-order periodicities or proximal-distal patterning. Leveraging the genetic tractability of trypanosomatid species, we quantified the location and contribution of each structure-identified protein to swimming behavior. Our study shows that proper B-tubule closure is critical for flagellar motility, exemplifying how integrating structural identification with systematic gene knockout can dissect individual protein contributions to flagellar motility.

Trypanosomatids are a family of parasitic protists that includes *Leishmania, Crithidia*, and *Trypanosoma* species and belong to the class Kinetoplastida. They are of medical importance as the causative agents of the human diseases leishmaniasis, trypanosomiasis, and Chagas disease. Members of this family rely on flagella for movement and survival in either their vertebrate host or insect vector. For example, the flagellated promastigote form of *L. mexicana*, which inhabits blood-feeding phlebotomine sand flies, requires flagellar motility to migrate from the insect midgut to the mouthparts (1), a critical step in enabling the parasite to be transferred to a mammalian host during feeding. Similarly, *Trypanosoma brucei* requires flagella for locomotion through the tsetse fly vector (2) and inhibition of motility in the host bloodstream is lethal for the parasite (3–5).

The beating movement of flagella is determined by the axoneme, an intraflagellar cylindrical arrangement of dynein-bound doublet microtubules (DMTs) that run the length of the flagellum. Cryo-EM has recently enabled molecular reconstructions of DMTs from a variety of single-celled organisms including *Chlamydomonas reinhardtii* (6, 7), *Tetrahymena thermophila* (8), and *Trichomonas vaginalis* (9), as well as from various mammalian ciliated cells (7, 10–13). These studies identify the proteins and complexes that adorn the luminal and external surfaces of DMTs in periodicities ranging from 8 to 96 nm. However, the specific contributions of many of these factors to flagellar motility remains unknown because current functional studies are incomplete, piecemeal, and often conducted in different organisms, hindering direct comparison.

Despite the overall conservation of the axoneme, clade-specific specialization of DMTs occurs. For example, *T. brucei* DMTs have unusual intraluminal densities in the B tubule called *ponticuli* (14), unexpected gaps in the microtubule lattice (15), and a morphologically distinct outer dynein arm docking complex (ODA-DC) compared to other species (15). Elucidating the molecular composition of these trypanosomatid-specific features, a prerequisite for understanding their function, requires high-resolution structural information.

## Structure determination of trypanosomatid doublet microtubules

**Figure 1:**
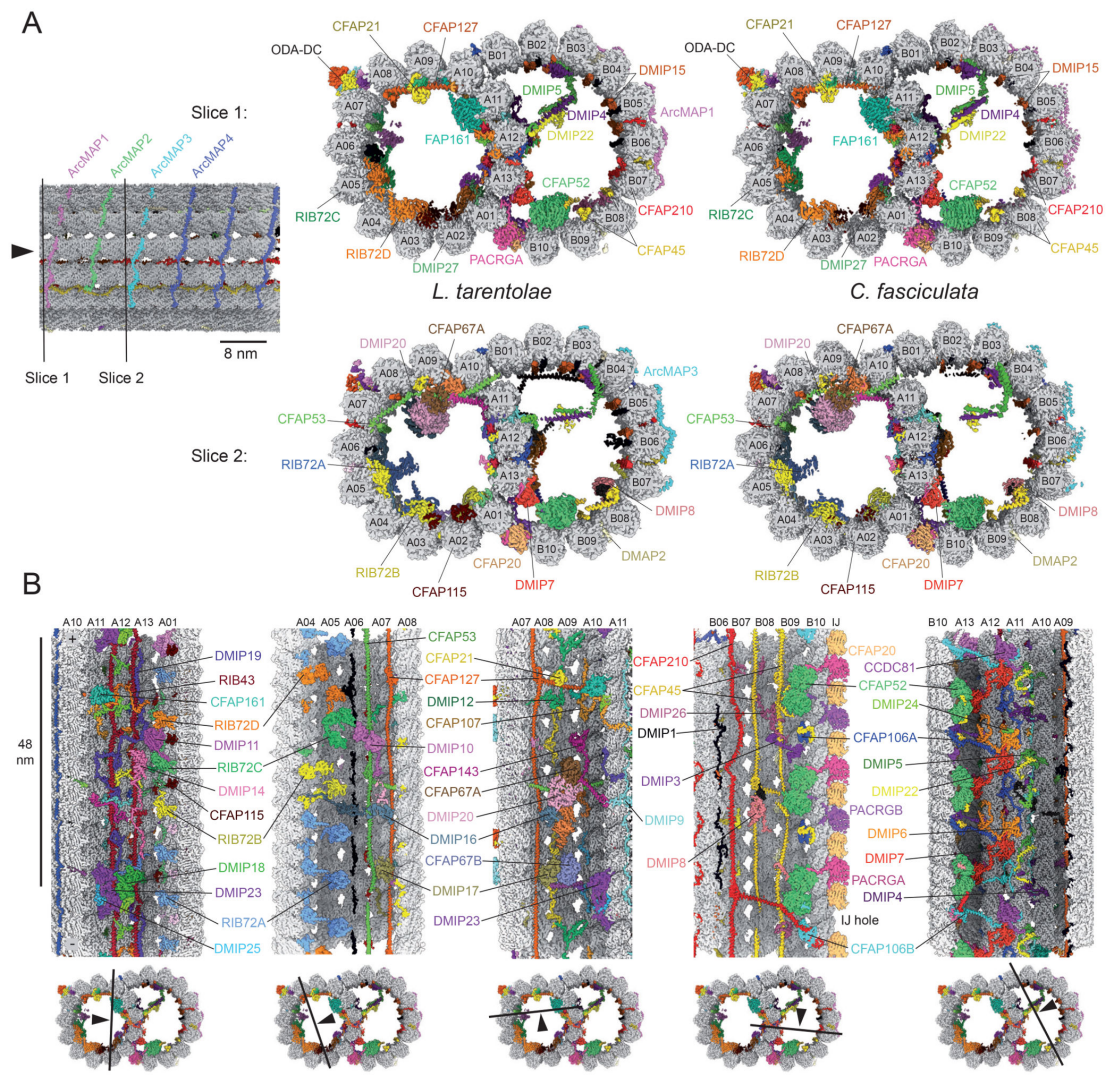
Structures of the 48-nm repeat of doublet microtubules (DMTs) from *L. tarentolae* and *C. fasciculata*. (**A**) Side-by-side comparison of two different slices through the DMT showing that all identified proteins are present in both the *L. tarentolae* and *C. fasciculata* structures. (**B**) Longitudinal slices through the cryo-EM map of the *L. tarentolae* DMT. Each microtubule inner protein (MIP) is given a unique color and labeled.

To resolve structures of DMTs from the trypanosomatid family, we used two non-human infective model organisms, dixenous *L. tarentolae*, which infects reptiles, and monoxenous *C. fasciculata*, which parasitizes only culicids. For each species, DMTs were obtained through splaying of demembranated axonemes prepared from mechanically isolated flagella (see materials and methods). Individual DMTs were analyzed by cryo-EM and processed using protocols for reconstructing DMTs at increasingly higher periodicities (16) (fig. S1-S2). This approach allowed us to resolve both the 48- and 96-nm repeats of the DMT (Table S1). The 48-nm repeat, the focus of this report, provides information on the MIPs and ODA-DC (Fig. 1), which have periodicities of 48 nm or less.

The 48-nm maps revealed densities corresponding to MIPs not observed in other species, as expected from the subtomogram averages of the *T. brucei* DMT (15, 17). To enable confident identification of these proteins, we used mask-focused local refinement to improve the resolution of each protofilament subregion to 2.7 to 3.3 Å (fig. S1-2). Within this resolution range, AI-based approaches for protein identification, such as ModelAngelo, have been shown to be highly successful (18). Initially, ModelAngelo was applied to the *L. tarentolae* DMT map, with the *C. fasciculata* map used for independent validation. In most cases, ModelAngelo identified homologous proteins when run independently on the *L. tarentolae* and *C. fasciculata* DMT maps, providing confidence that a correct solution had been found (fig. S3). ModelAngelo was also able to distinguish between paralogous proteins (fig. S4). Each solution was also evaluated by (i) manually inspecting the fit of the sidechains to the density (fig. S5-S15), (ii) comparing the ModelAngelo-built model with the AlphaFold2 (AF2) prediction (fig. S3), and (iii) confirming the presence of the protein in mass spectrometry analysis of purified flagella (Data S1). This approach enabled us to identify 51 MIPs, 11 MAPs, and a 5-subunit ODA-DC (Data S2).

The *L. tarentolae* and *C. fasciculata* DMTs have identical MIP repertoires (Fig. 1), with ∼87% of all MIP residues being identical between the two species (fig. S16). Each protein also has an ortholog in *T. brucei*, with an average identity of 45% between *L. tarentolae* and *T. brucei* homologs (fig. S16 and Data S2), supporting the notion that trypanosomatid axonemes are highly conserved. This analysis is consistent with comparative phylogenetics indicating that *Leishmania* and *Trypanosoma* separated 190 million years ago (MYA), while *C. fasciculata* branched off from *Leishmania* more recently (109 MYA) (19). Given the high degree of structural similarity, we focused our analysis on the *L. tarentolae* DMT.

## Analysis of protein conservation

**Figure 2:**
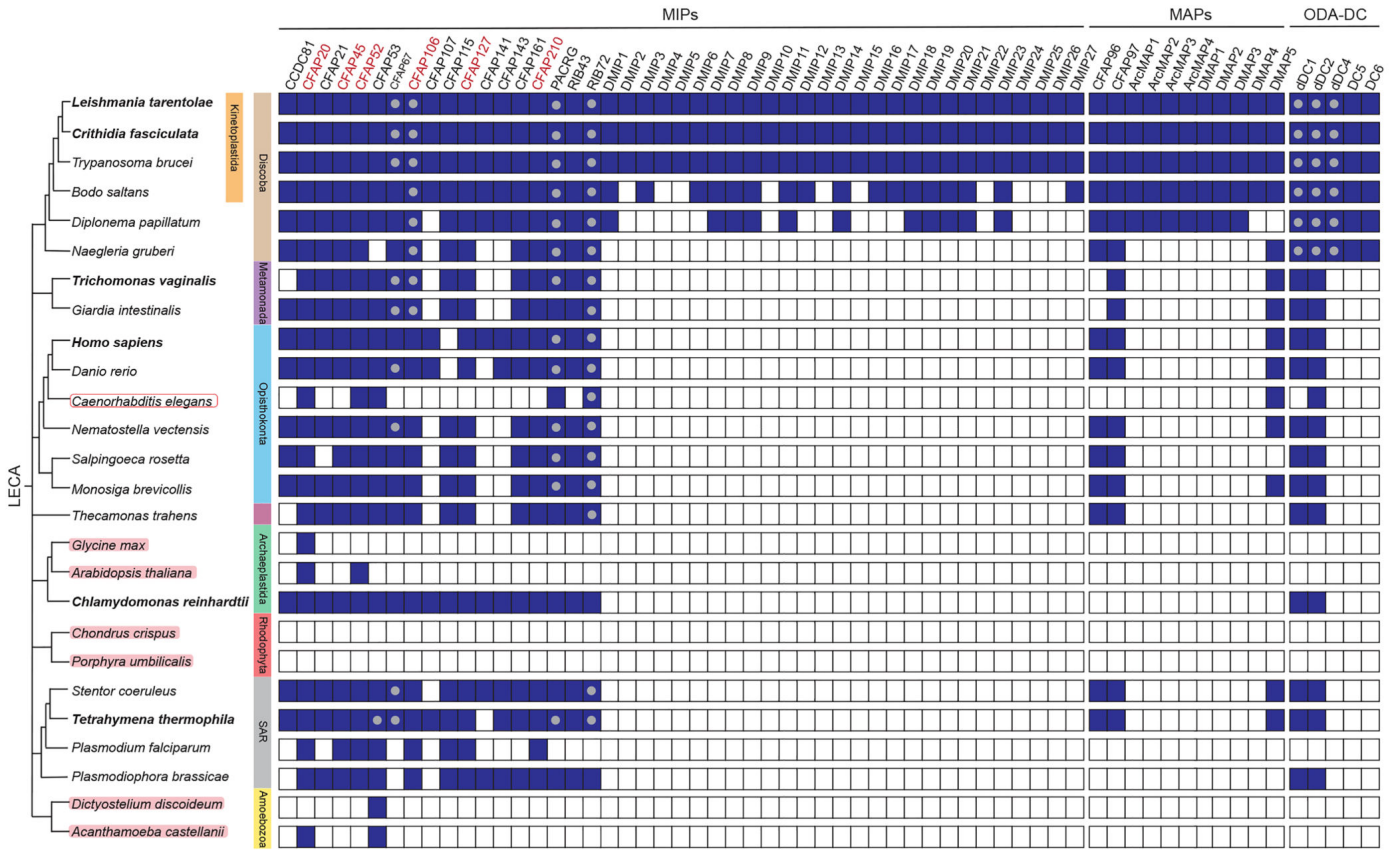
Evolutionary distribution of doublet microtubule associated proteins. A table showing the presence (blue squares) or absence (white squares) of orthologs of proteins identified in this study. Species are ordered according to a stylized eukaryotic tree (left column). Species with structurally resolved doublet microtubules are emphasized in bold, while those lacking cilia/flagella are highlighted in red. *C. elegans* (boxed in red) only builds non-motile cilia. Grey circles denote the presence of more than one putative ortholog. Proteins are organized into three classes: microtubule inner proteins (MIPs), microtubule associated proteins (MAPs) and subunits of the outer dynein arm docking complex (ODA-DC). Proteins found in all examined species possessing motile flagella are named in red.

To evaluate the evolutionary conservation of each identified DMT-associated protein, we used sequence- and structure-based bioinformatic approaches (see materials and methods) to search for orthologs in well-studied ciliated organisms, including all those with existing DMT structures, and a few model organisms that either lack motile cilia or cilia entirely (Fig. 2). Just six MIPs have orthologs in all motile-ciliated organisms examined. Whether these are universal to DMTs is difficult to ascertain given the potential for divergent axonemes in organisms not yet studied. Nonetheless, because the analyzed species encompass most major clades, these proteins likely represent a minimal set of “core MIPs” needed for proper DMT stability and function in motile cilia. The core MIPs include CFAP20, which connects the A and B tubules at the inner junction (IJ), CFAP52 and CFAP106, two proteins that bind near the IJ, and three filamentous MIPs (fMIPs; CFAP45, CFAP127, CFAP210) that bind interprotofilament clefts through long α-helices and help establish the overall 48-nm periodicity of DMT interiors (6, 20). CFAP161, RIB43, RIB72, and PACRG are present in all motile ciliated species examined except *Plasmodium falciparum*, the flagellated parasite responsible for malaria. *Plasmodium* DMTs are notable for their short lifespans and intraflagellar transport (IFT)-independent assembly (21, 22), suggesting that CFAP161, RIB43, RIB72, and PACRG may be required for the stability of axonemes with a life span exceeding a few minutes or IFT-based assembly.

We also identified 27 MIPs not observed in previous DMT structures that we named DMIPs (doublet microtubule inner proteins). Ten of these are specific to trypanosomatids, six are confined to kinetoplastids, and 11 have homologs in Discoba (Fig. 2). Many of the DMIPs have no clearly defined globular domain and likely play structural roles by binding tubulin and other MIPs. DMIP15 is a previously unannotated fMIP. Eight DMIPs contain EF-hand motifs and are therefore potentially capable of binding calcium. Many algal-specific MIPs also contain EF-hand motifs but are bound in different locations (6), suggesting that calcium-binding proteins have been added to DMTs independently in different lineages following the Last Eukaryotic Common Ancestor (LECA). Four DMIPs also have zinc-binding sites. Of the five newly identified external doublet microtubule-associated proteins (DMAPs), only DMAP5 has a taxonomic distribution outside discoba (Fig. 2).

## Systematic analysis of location and asymmetry

**Figure 3:**
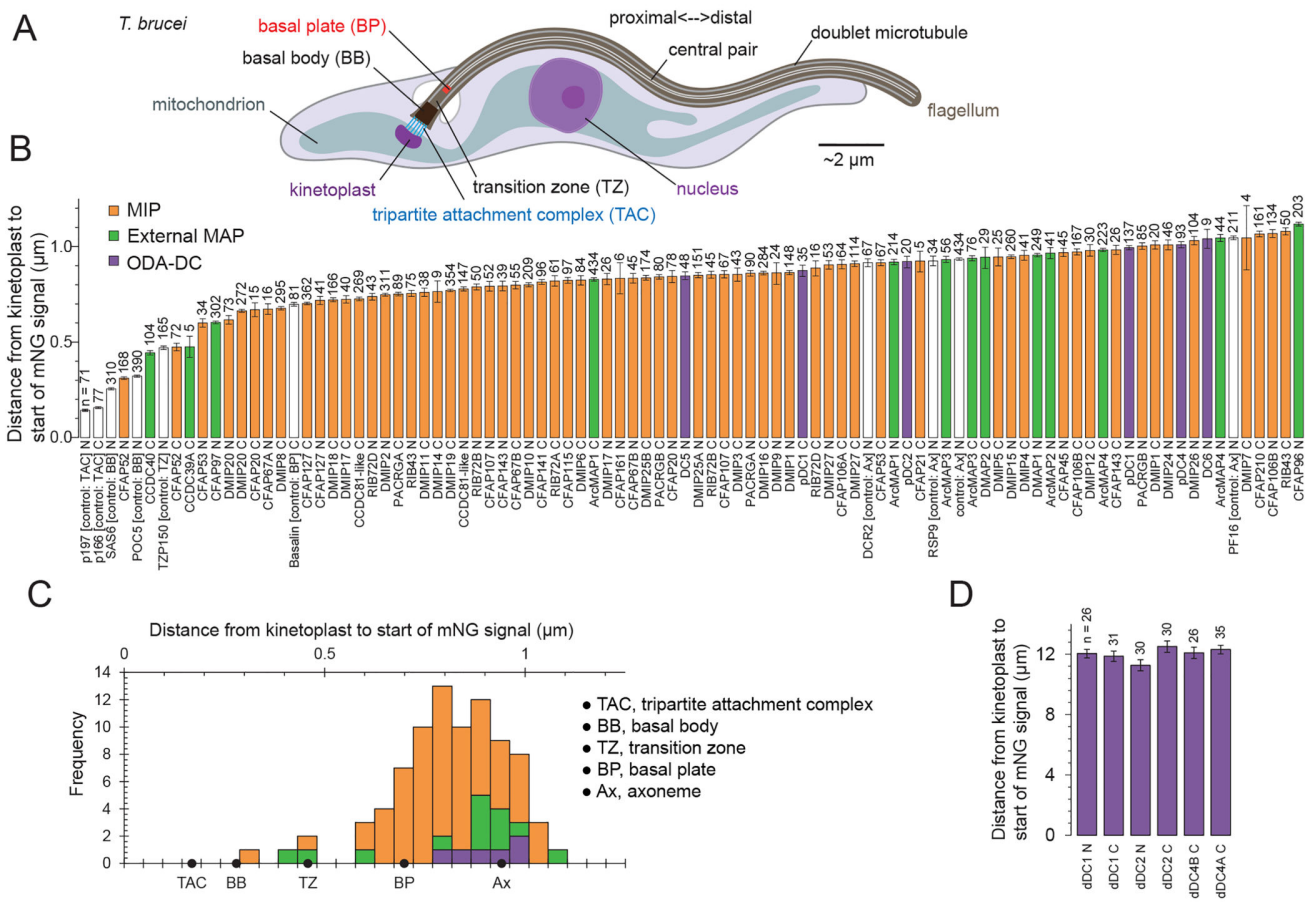
Localization data for the identified DMT-associated proteins. (**A**) A diagram showing the morphology of the procyclic form of *T. brucei*. (**B**) Automated measurement of the distance from the centroid of the kinetoplast DNA stain signal to the start of the mNeonGreen (mNG) fluorescence signal. The graph shows mean and standard error, with number of cells measured shown at the top of each bar. N and C terminally tagged cell lines were measured independently. Proteins are classified as microtubule inner proteins (MIPs, orange), external microtubule-associated proteins (MAPs, green) or subunits of the outer dynein arm docking complex (ODA-DC, purple). Controls are shown in white: p166 and p197 are components of the tripartite attachment complex (TAC); SAS6 and POC5 are components of the basal body (BB); TZP150 is a component of the transition zone; Basalin marks the basal plate (BP); DRC2, ODAα, RSP9 and PF16 are components of the axoneme. (**C**) A histogram summarizing the localization data shown in panel A. The majority of signals start between the basal plate (BP, defined by Basalin) and the axoneme (Ax, defined by the average of DRC2, ODAα, RSP9 and PF16). (**D**) Manual measurement of the distance from the kinetoplast to the distal ODA-DC subunits. Note that *T. brucei* has two dDC4 paralogs (dDC4A and dDC4B) due to chromosome 4/8 duplication.

Because our samples are sheared from the proximal end (∼1 μm) of the flagellum by mechanical separation, our structures are biased toward identifying proteins present in distal DMTs. Given the potential for axonemal proteins to display proximal-distal asymmetry or localize elsewhere in the cell, we evaluated the location of every protein identified in this study systematically within live cells. To do this we utilized TrypTag, a database of fluorescence imaging generated by endogenously fusing a mNeonGreen fluorescent marker onto the N- and/or C-termini of almost every protein in the procyclic form of *T. brucei* (23, 24). All proteins identified in our study have an ortholog in *T. brucei* (Fig. 2) and have corresponding images in TrypTag (fig. S17-S18), consistent with their trypanosomatid-wide distribution. We developed an automated method to quantify the onset of the mNG fluorescence signal in relation to the position of the kinetoplast DNA specifically in cells with a single kinetoplast and nucleus (1K1N cells). For comparison, we also quantified proteins known to localize to the tripartite attachment complex, the basal body, the transition zone, the basal plate, or the axoneme (see Fig. 3 and materials and methods). Our analysis revealed that all identified MIPs are also present in the proximal axoneme (Fig. 3B and fig. S17) and that there is no significant difference in the onset of signals for the conserved MIPs and the newly identified DMIPs. Consequently, our structures are likely representative of both the proximal and distal parts of the axoneme. Our analysis further indicates that the signal for most MIPs first appears after the basal plate but before the major external axonemal complexes and the central pair. This pattern implies that the 48-nm MIP network is established prior to the formation of the external 96-nm repeat. The absence of axonemal MIPs in the basal body and transition zone – with the notable exception of CFAP52 – challenges the hypothesis that a subset of MIPs establishes the periodicity of the axoneme in the basal body (6). It also suggests that MIPs present in the basal body and transition zone differ from axonemal MIPs, with CFAP52 being the sole exception.

In the *T. brucei* cell cycle, the duplication and segregation of the kinetoplast (K) and nucleus (N) follows a defined order, with the number of K and N indicating how far along the cell cycle has progressed. In the 2K1N phase, cells assemble a new flagellum posterior to the old one. We utilized this phenomenon to analyze 2K1N cells in the TrypTag imaged population for proteins identified in our structure that display an asymmetric distribution between old and newly formed flagella. Our findings reveal that most MIPs show no preference and are present in both flagella. However, distinct cell-cycle stage differences were observed for four proteins (fig. S19). Specifically, DMAP2 and DMIP21 were enriched in new flagella (fig. S19A), whereas DMIP4 and DMIP5 were enriched in old flagella (fig. S19B). The discovery of proteins that preferentially associate with new flagella was surprising, given that our structures are averages of all flagella, which will be biased towards mature flagella. We therefore tagged all four proteins (DMIP4, DMIP5, DMIP21 and DMAP2) with mNeonGreen in *L. mexicana* (Table S2) and analyzed their localization in mature and growing flagella (fig. S20A-C). No robust signal was detected for DMIP4 following tagging, but DMIP5 was confirmed to be specific for mature, long flagella with the signal confined to the distal end of the flagellum (fig. S20A). Unlike in *T. brucei*, DMIP21 and DMAP2 were present in both old and new flagella (fig. S20B-C), consistent with their presence in our structure. Growth of the new flagellum occurs earlier in the *T. brucei* than *L. mexicana* cell cycle (25), which may indicate a biological difference. Alternatively, it may reflect an artifact of tagging where the timing of incorporation of the protein into the axoneme is altered by the tag interfering with native interactions in the cell or by the replacement of endogenous genetic elements critical for cell cycle regulation through the tagging process.

## Most MIPs are dispensable for swimming

**Figure 4:**
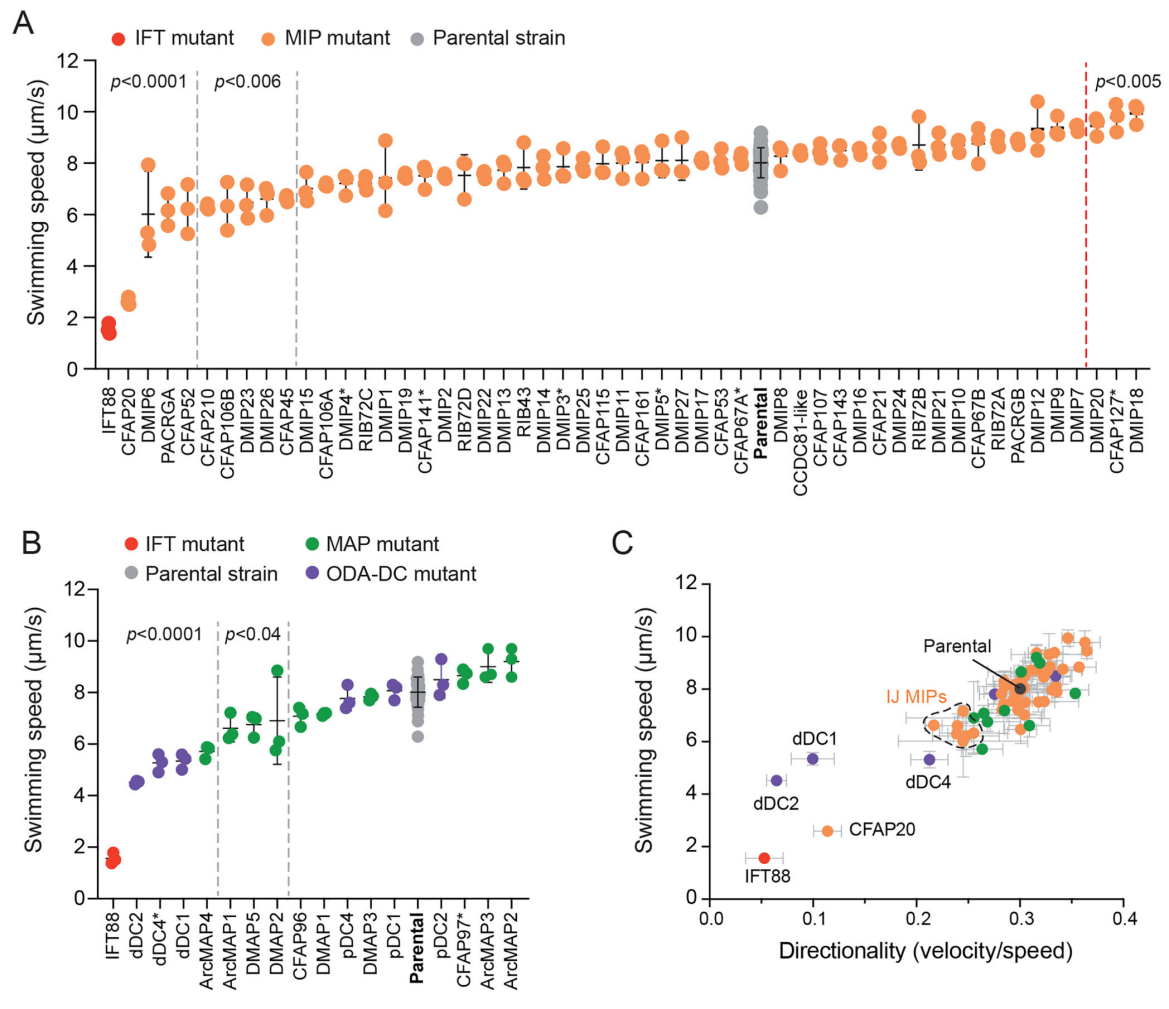
Motility phenotypes of *L. mexicana* knockout mutants. (**A**) Swimming speed of microtubule inner protein (MIP) deletion cell lines. (**B**) Swimming speed of external microtubule-associated protein (MAP) deletion cell lines. For each cell line, the measurements from each replicate are shown as dots and the bars show the mean and standard deviation. The parental cell line (*L. mexicana* Cas9 T7; grey dots) and a non-motile Δ*IFT88* mutant (red dots) were used as controls. Mutants before the grey dashed lines have statistically significant reductions in swim speed at the *p*-values indicated (one-way ANOVA with Dunnett’s multiple comparisons test). Mutants after the red dashed line have a statistically significant increase in swim speed. Asterisks indicate incomplete gene deletions. (**C**) The mean swimming speed of all MIP and MAP knockout mutants are plotted against mean directionality (the ratio of velocity to speed). Bars represent the standard deviation of the three replicates for each mutant. The parental cell line (*Lm*Cas9 T7; grey) and the non-motile Δ*IFT88* mutant (red dot) were used as controls. A cluster of inner-junction (IJ) proteins with reduced swim speed and directionality are highlighted.

The genetic tractability of *Leishmania* species next allowed us to examine the individual contribution of each identified axonemal protein to ciliary motility. Using CRISPR-based gene editing (26, 27) we engineered knockouts of each protein in *L. mexicana*, a species closely related *to L. tarentolae*. We did not obtain knockouts for two proteins and eight others were incomplete deletions where at least one percent of the population retained a copy of the gene (labeled with asterisks in Fig. 4).

For each of the 67 knockout mutants generated, we used darkfield videomicroscopy to measure mean speed and directionality. We conducted three measurements for each cell line under identical conditions and analyzed the movies using automated methods (1, 28) to provide quantitative and comparative data (Data S3). As controls, we also analyzed the swimming behavior of the parental strain and a non-motile IFT88 knockout mutant that did not assemble flagella (1). Qualitative data were also recorded for the growth rate of the mutant, its morphology and flagellum length (Data S3).

Our data indicates that most MIPs are not essential for swimming. Only nine MIPs showed statistically significant (*p*<0.006, one-way ANNOVA with Dunnett’s multiple comparisons test) reductions in swim speed (Fig. 4A). Six of these belong to core or highly conserved MIP categories (CFAP20, CFAP45, CFAP52, CFAP106B, CFAP210, and PACRGA) and three are specific to trypanosomatids (DMIP6, DMIP23 and DMIP26). All but DMIP23 cluster at the IJ and interact with one another, suggesting that proper closure of the B tubule is important for axoneme structure and function. Despite being similarly located, the paralogous proteins CFAP106A and PACRGB did not significantly reduce swimming speed, suggesting they have redundant functions. Knockout of CFAP20 was particularly severe, causing almost complete paralysis, and upon closer inspection, an absence of long flagella. A similar phenotype was observed following CFAP20 knockdown in *T. brucei*(29), suggesting that CFAP20 is essential for flagella assembly within the trypanosomatid family. In other species, CFAP20 loss is better tolerated (30, 31).

Roles for the other conserved IJ proteins in DMT stability and/or flagella motility are supported by prior work in other species. For example, knockout of CFAP45 or CFAP52 in *C. reinhardtii* results in B-tubule instability (31), knockdown of CFAP106 in *T. brucei* produces short flagella and aberrant motility (17) and abnormal sperm motility in mice (32). Knockout or knockdown of PACRG results in sterility in mice (33), defects in left-right asymmetry in vertebrates (34) and defective axonemes with missing DMTs in *T. brucei* (35).

DMIP23 is the only non-IJ MIP whose mutant displayed a significant reduction in swim speed (*p*=0.001) and directionality. This protein has a β-trefoil domain resembling CFAP161, an EF hand domain, and protrudes an α-helical extension through the A-tubule seam to the external side of the DMT. The mechanism by which loss of this protein reduces swim speed is unclear. Three mutants (ΔDMIP18, ΔDMIP20 and ΔCFAP127) had a small but statistically significant increase in swim speed (*p*=<0.005).

**Figure 5:**
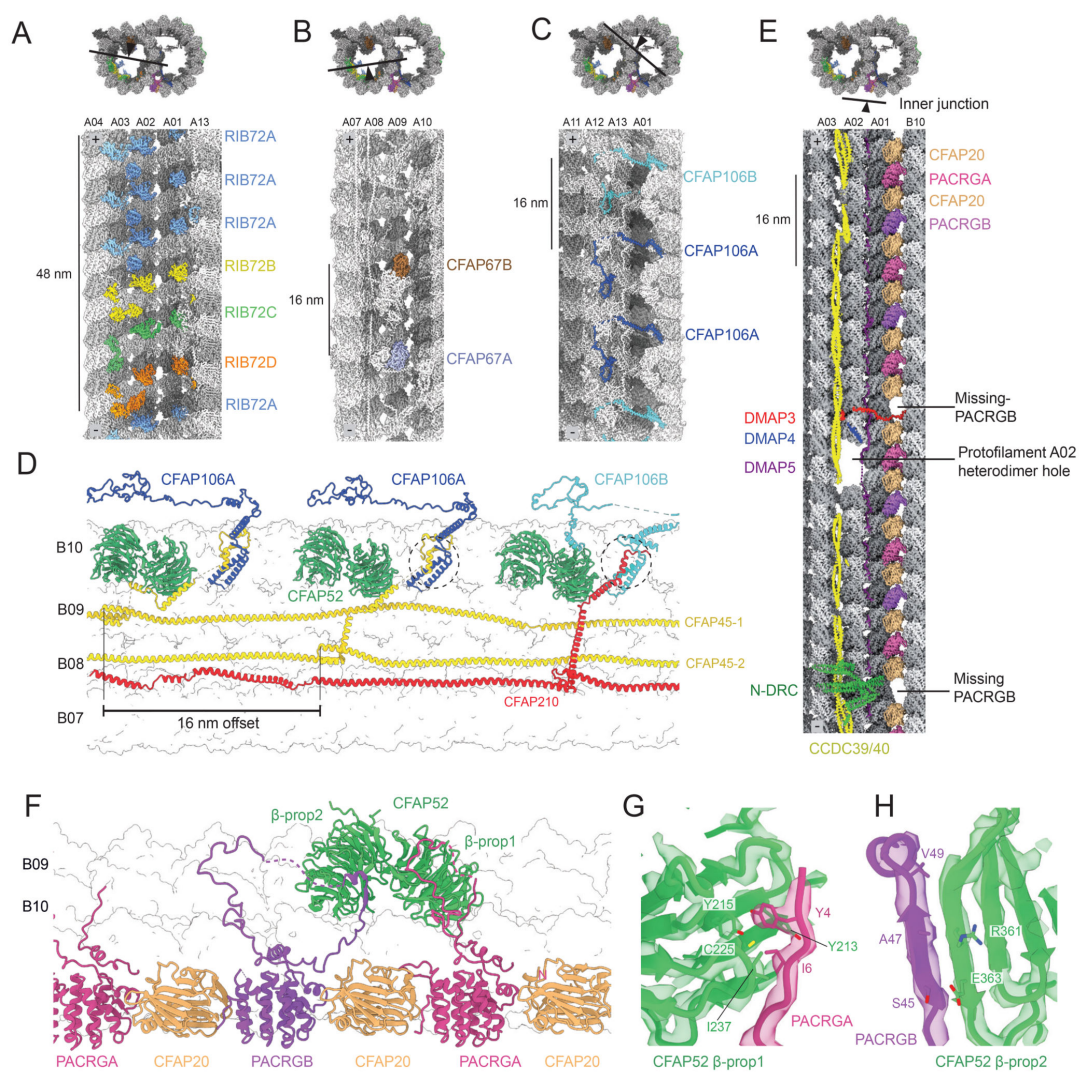
Paralogous microtubule inner proteins of the *L. tarentolae* DMT. (**A**) Cross-sectional and longitudinal view of the organization of RIB72 paralogs in the A tubule. (**B**) Positions of CFAP67 paralogs. (**C**) Repeat pattern of CFAP106 paralogs. (**D**) Repeat pattern of CFAP106A and CFAP106B and their relationship to the filamentous MIPs, CFAP45 and CFAP210. **(E**) Cross-sectional and longitudinal view of the inner junction showing the repeat pattern of CFAP20 with PACRG paralogs and the presence of holes in the microtubule lattice. (**F**) The repeating pattern of PACRGA and PACRGB is determined by CFAP52. (**G**) Details of the interaction between PACRGA and CFAP52. (**H**) Details of the interaction between PACRGB and CFAP52.

**Figure 6:**
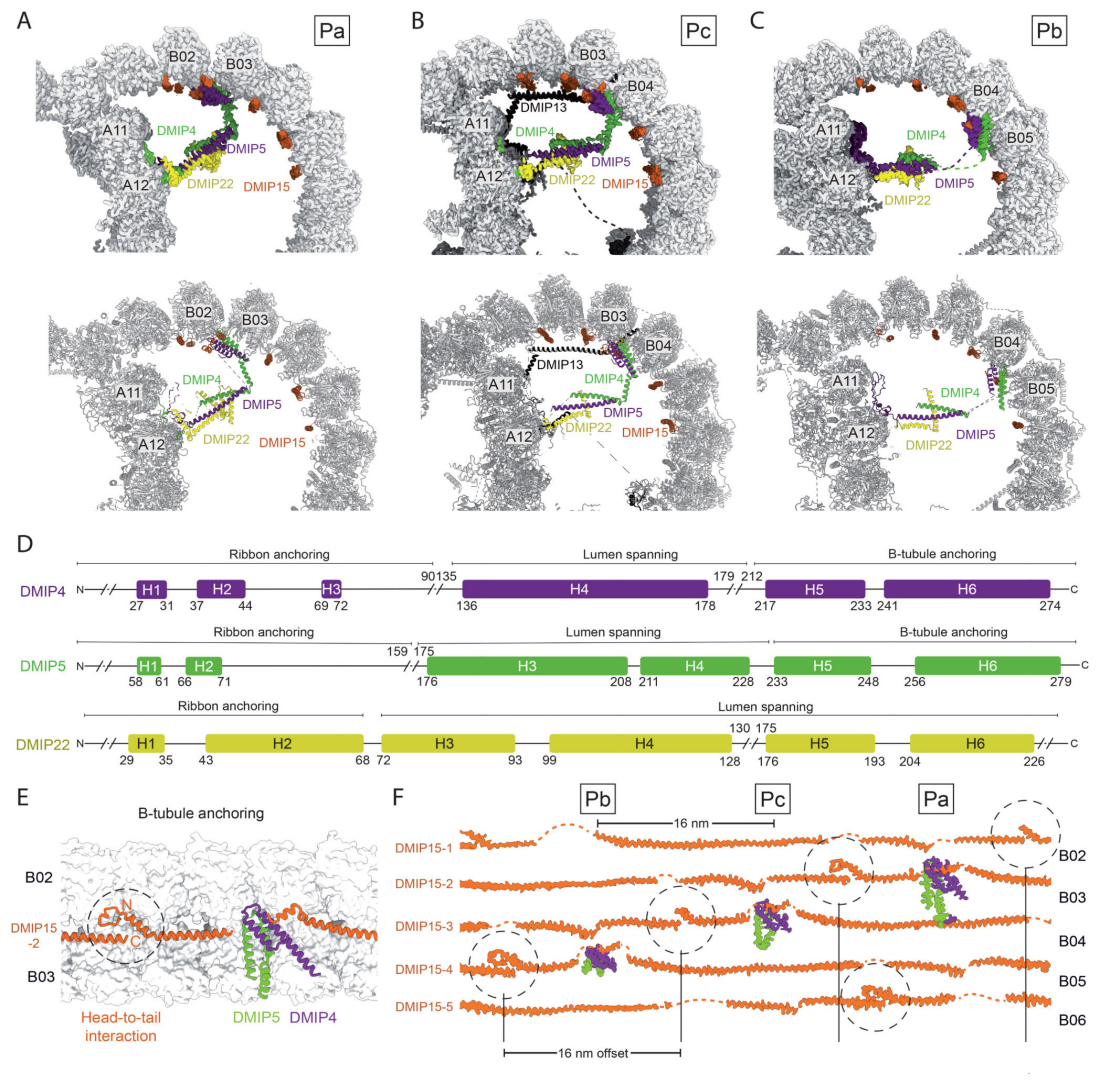
Structural basis of the *ponticulus*. (**A-C**) Cryo-EM maps (top) and atomic models (below) for the three different conformations of the *ponticulus* in *L. tarentolae*. These conformations are labeled Pa, Pb, and Pc, following the naming scheme established in (15). DMIP13 is a *ponticulus*-associated protein that binds only Pc. (**D**) Secondary structure diagrams of the three *ponticulus* subunits. (**E**) Atomic model showing that DMIP4/5 engages the B tubule in a site determined by DMIP15 (orange). (**F**) The staggered offset of multiple DMIP15 proteins on neighboring B-tubule protofilaments determines the 16-nm spacing of the *ponticuli*.

**Figure 7:**
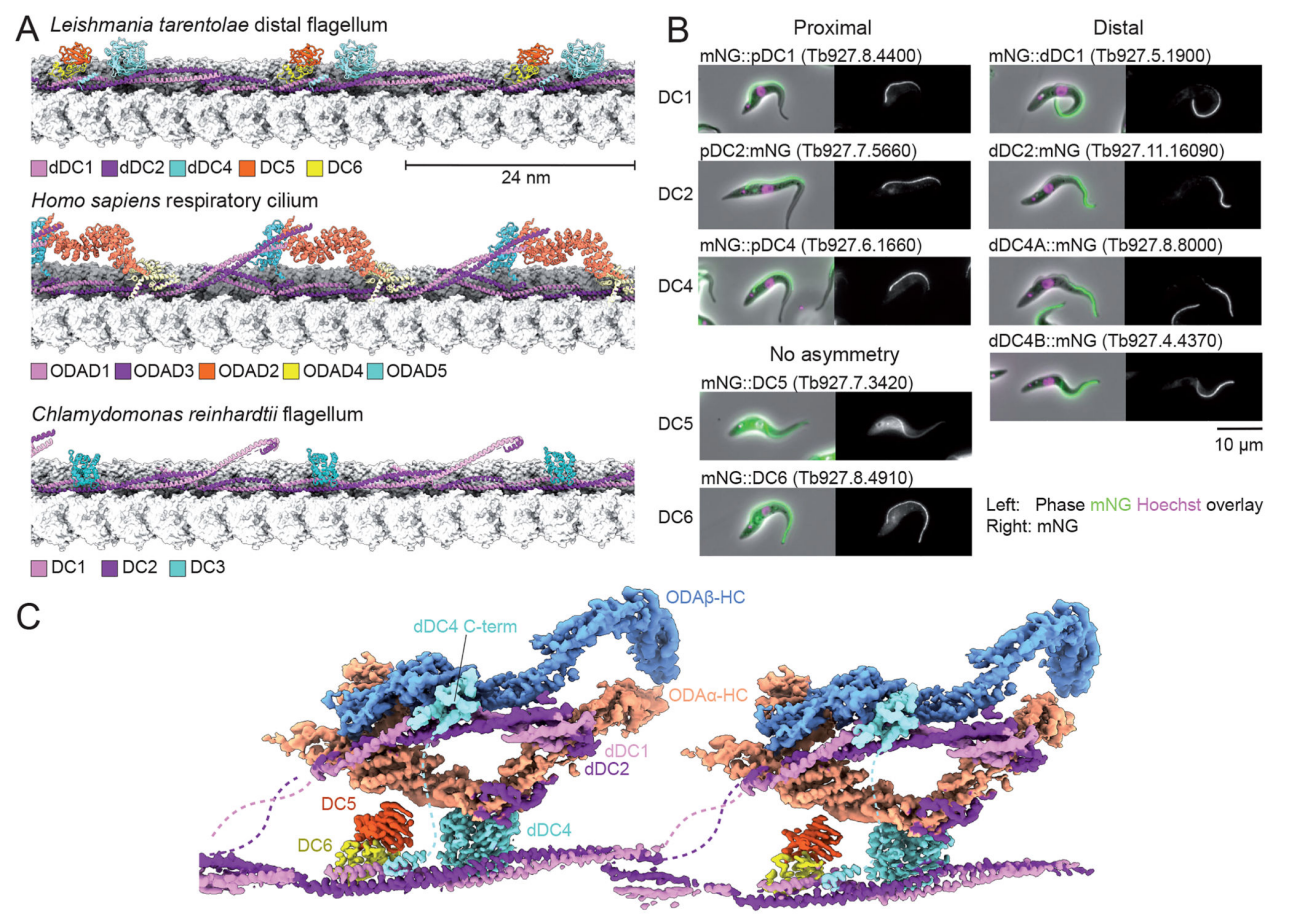
The *L. tarentolae* outer dynein arm docking complex (ODA-DC). (**A**) Comparison of the atomic model of the distal *L. tarentolae* ODA-DC with the ODA-DCs of human respiratory cilia (PDB: 7UNG) (11) and *C. reinhardtii* flagella (PDB: 6U42) (6). Tubulin protofilaments A07 and A08 are shown in surface representation. (**B**) Fluorescence signal distribution for *T. brucei* ODA-DC subunits endogenously tagged with N- or C-terminal mNeonGreen (mNG). Phase contrast (grey), DNA (Hoechst 33342, magenta) and mNG (green) overlay (left) and mNG fluorescence (right) are shown. DC1, 2 and 4 have paralogs that show clear proximal-distal asymmetries. DC5 and DC6 are present throughout the flagellum. Note that unlike *Leishmania* species, *T. brucei* has two dDC4 paralogs (dDC4A and dDC4B) resulting from chromosome duplication. (**C**) Composite cryo-EM map showing the interaction between two neighboring ODAs and their docking complexes. Some ODA subunits have been hidden for clarity.

Below we use our systematic analysis of DMT protein structure, evolution, location, and function (Fig. 1–4) to illustrate three examples where trypanosomatid DMTs have diverged from others: the emergence of complex repeat patterns through paralogous proteins (Fig. 5), the appearance of B-tubule *ponticuli* (Fig. 6), and the adaptation of the dynein-docking machinery (Fig. 7).

## Paralogous proteins generate complex repeat patterns

In trypanosomatids, several conserved MIPs (CFAP67, CFAP106, PACRG, and RIB72) have two or more paralogs (Fig. 2). In each case, our structures unequivocally demonstrate that these paralogs bind within a single 48-nm repeat, each exhibiting a unique pattern of repetition (Fig. 5A-E and fig. S4). Their co-existence along the length of the flagellum is supported with TrypTag localization data (fig. S18). The presence of multiple paralogous proteins within a single repeat generates a novel periodic pattern compared to those in previous DMT structures, increasing the overall complexity of the MIP network.

CFAP67 paralogs and PACRG paralogs repeat in a simple A-B-A-B pattern, dictated by their interacting MIPs. For example, the incorporation of PACRGA and PACRGB into the IJ with alternating 16-nm periodicity appears to be determined by the interactions their divergent N-termini make with different β-propeller domains of CFAP52 (Fig. 5F). The repeat patterns for RIB72 and CFAP106 are more complex. The four paralogous RIB72 proteins (designated A-D) bind with a A-A-A-B-C-D pattern within a 48-nm section of the A tubule with a paralog every 8 nm (Fig. 5A), whereas the two paralogs of CFAP106 bind with a A-A-B pattern with a paralog every 16 nm (Fig. 5C). In the case of CFAP106, the patterning is determined by an offset arrangement of fMIPs bound to three adjacent B-tubule interprotofilament clefts (Fig. 5D). Specifically, there are two copies of CFAP45 and one copy of CFAP210, each offset from its neighboring fMIP by 16 nm. The selective binding of CFAP106A to the C-terminal regions of the two CFAP45 proteins, and CFAP106B binding to the C-terminus of CFAP210, generates the repeating A-A-B order. This mechanism therefore creates complex repeating patterns from offset 48-nm proteins and demonstrates the pivotal role that fMIPs play in establishing periodicity within DMTs.

The pattern of CFAP106 paralogs also explains two gaps in the IJ filament (Fig. 5E), an unusual feature first observed by subtomogram averaging of *T. brucei* axonemes (15). Our structures show that both holes correspond to absent PACRGB subunits, and that the pattern of loss corresponds with the presence of CFAP106B. Sites where PACRGB is retained contain the CFAP106A-interacting protein, DMIP6 (LtaP24.0150), which interacts with variable PACRGB N-terminus. A role for DMIP6 in maintaining proper IJ organization is supported by a statistically significant reduction of swim speed and directionality in ΔDMIP6 mutants (Fig. 4). Consistent with a model where the position of IJ holes is dictated by the MIP network, knockdown of the *T. brucei* ortholog of CFAP106A causes reduction in the levels of the DMIP6 ortholog and the appearance of six holes per 96-nm repeat (17), presumably corresponding to the loss of all PACRGB subunits.

Both *L. tarentolae* and *C. fasciculata* DMT structures also exhibit a gap in protofilament A02 that corresponds to an absent tubulin heterodimer (Fig. 5E). The molecular basis for this gap, which also appears in a subset of DMTs from the choanoflagellate *Salpingoeca rosetta* (36), is unclear. A potential role for DMAP4 and DMAP5, which are adjacent to, or even partially within the hole (Fig. 5E), is not supported by their absence from *S. rosetta* (Fig. 2).

Paralogous proteins with complex repeat patterns are not confined to the DMT lumens. We also observed a class of four paralogous ArcMAPs, named because of the lateral arc they form across the exterior of protofilaments B04-B08 (fig. S21). ArcMAPs are characterized by at least four helices (fig. S21B), with each helix interacting with successive protofilaments often through a conserved arginine that forms a salt bridge with D431 of α-tubulin (fig. S21D, E). The ArcMAPs displayed a 1-2-3-4-4-4 pattern of periodicity, with each ArcMAP separated longitudinally by 8 nm (fig. S21A). The mechanism driving this pattern remains elusive, because interactions with other MAPs or MIPs are minimal. In our knockout studies, deletion of ArcMAP1 and ArcMAP4 caused a statistically significant reduction in swim speed, whereas knockout of ArcMAP2 and ArcMAP3 did not (Fig. 4B). These results indicate that while ArcMAP1 and 4 play a role in motility through an unknown mechanism, ArcMAP2 and 3 are redundant. We note that the unrelated proteins SPMAP2 and SPMAP2L occupy a similar structural niche as the ArcMAP paralogs in mammalian sperm DMTs (12) (fig. S21C).

## B-tubule *ponticuli*

A defining feature of trypanosomatid DMTs are repetitive densities that stretch across the B-tubule lumens termed *ponticuli*, from the Latin for “little bridges” (14). These *ponticuli*, as revealed by stained, thin-section transmission electron microscopy, are specific to mature axonemal DMTs (14). Cryo-ET and subtomogram averaging of *T. brucei* DMTs further revealed 3 discrete *ponticuli* within a 48-nm repeat, named Pa, Pb, and Pc (15). These are spaced 16 nm apart and are identifiable by their attachment to different protofilaments of the B tubule (B3, B4, or B5) but the same protofilament of the A tubule (A12).

Contrary to expectations that each *ponticulus* corresponds to a different MIP (15), we found that each is formed by a heterotrimeric complex of DMIP4, DMIP5 and DMIP22 (Fig. 6A-C). *Ponticulus* Pc is further bound by DMIP13, a lumen-spanning *ponticulus*-associated protein (Fig. 6B). Given that DMIP4 and DMIP5 are adjacent on the genome and have similar secondary structure profiles (Fig. 6D), we infer that they are paralogs resulting from a recent gene duplication event. Together, these proteins form an elongated and predominantly α-helical complex (Fig. 6A-C). Flexible loops connecting the helices provide the plasticity necessary to bridge multiple sites of the B tubule.

The ability of the *ponticulus* heterotrimer to recognize three different sites of the B tubule is determined by the association of DMIP4/5 with DMIP15, a 48-nm B-tubule fMIP that occupies the five adjacent interprotofilament clefts between protofilaments B01 and B06 (Fig. 6E). These five copies run parallel to one another but with adjacent chains offset by 16 nm. Owing to the offset of multiple copies of DMIP15, binding sites for DMIP4/5 appear with 16-nm periodicity on adjacent protofilaments (Fig. 6F). Similar to the CFAP106 example above, this demonstrates how offset 48-nm fMIPs provides a mechanism to create complex repeating patterns for interacting MIPs.

Prior work has established that *ponticuli* only appear on axonemes of mature kinetoplastid flagella (14, 37). Our analysis of 2K1N cells in TrypTag supports this observation (fig. S19C). We observed preferential association of the *ponticulus*-associated proteins, DMIP4 and DMIP5, with old flagella, although the signal for DMIP22 is too weak to draw a similar conclusion. Notably, tagging the C-terminus of DMIP4 or DMIP5 in *T. brucei* disrupted the enrichment pattern (fig. S19D) presumably by either overwhelming or suppressing the mechanism that delays its incorporation into the axoneme. This suppression was not observed in *L. mexicana*, where C-terminally tagged DMIP5 displayed the same preference for mature flagella (fig. S20) as N-terminally tagged DMIP5 in *T. brucei* (fig. S19C).

### Trypanosomatid-specific dynein-docking subunits

Our structures demonstrate that trypanosomatids possess a five-subunit ODA-DC (Fig. 7A). Of these subunits, only two (LtaP15.0520 and LtaP32.3090) have equivalents in mammalian (10) and algal ODA-DCs (6). These subunits correspond to DC1 and DC2 which form a heterodimeric coiled coil situated in the cleft between protofilaments A07 and A08. Prior work has established that trypanosomatids have two paralogs of the DC1/2 heterodimer, one specific to the proximal axoneme (pDC1/2) and another to the distal axoneme (dDC1/2) (38). Given that our maps are predominantly generated by averaging distal DMTs, we observed only the distal paralogs (fig. S22).

Bound to the dDC1/dDC2 coiled coil is LtaP31.0090, a protein with structural similarity to ATP-grasp domain proteins. Despite occupying a similar binding site as *C. reinhardtii* DC3 (Fig. 7A), it shares no sequence or structural similarity. We therefore name it dDC4 and infer that its proximal paralog (LtaP30.0300) is pDC4 (Fig. 7B). Independent evidence supporting the claim that DC4 is an ODA-DC subunit with asymmetrically distributed paralogs comes from a recent study that exploited the TrypTag dataset to identify *T. brucei* flagellar proteins with similar proximal-distal distributions as DC1/DC2 (39).

The binding of dDC4 atop dDC1/2 suggests that both distal and proximal paralogs of DC4 rely on DC1/DC2 for their integration within the axoneme. This apparent dependency is supported by gene knockout studies conducted in *L. mexicana* (39). In the absence of dDC2, the distal axoneme signal for dDC4 is lost. Similarly, without pDC1, the proximal axoneme signal for pDC4 disappears.

By mining our cryo-EM dataset for particles with bound ODAs (fig. S23), we found that dDC4 interacts with the ODAα heavy chain (Fig. 7B). Loss of dDC4 caused a reduction in swim speed to the same levels as ΔdDC1 or ΔdDC2 mutants (Fig. 4B), suggesting that this interaction contributes to the anchoring of ODA to the axoneme. In contrast, loss of pDC4 or pDC1/2 had no negative effect on swim speed, consistent with observations made in a separate study (39).

The remaining trypanosomatid-specific ODA-DC subunits, DC5 (LtaP22.1400) and DC6 (LtaP16.1580), form a heterodimer that associates with the DC1/2 coiled coil through the DC6 subunit. DC5 is structurally similar to FKBP class peptidyl-prolyl isomerases (PPI) and DC6 is a zinc-binding protein. Both proteins lack obvious paralogs and TrypTag data indicates that they are present throughout the flagellum (Fig. 7C), suggesting that they may bind both pDC1/2 and dDC1/2. Consistent with this possibility, their binding site is conserved on both DC1/2 paralogs. We speculate that DC5/6 may function to stabilize the DC1/2 coiled coil because neither contacts the ODA.

Collectively, our findings point to the ODA-DC as a locus for adaptation, providing opportunities to duplicate or embellish the DC1/2 coiled coil with additional subunits to establish axonemal asymmetries or regulate dyneins.

## Discussion

Our structures of trypanosomatid DMTs reveal how gene duplication and emergence of paralogous proteins creates complexity by generating increasingly intricate patterns of periodicity or by promoting proximal-distal asymmetry. The prevalence of MIP paralogs across taxa suggests that the diversification of existing building blocks has enabled elaboration of DMTs in diverse lineages. One potential advantage of paralog expansion could be the provision of protective functional redundancy. This is supported by the observation that knockdown of both PACRG paralogs in *T. brucei* causes paralysis while knockdown of individual paralogs does not (35). Another potential evolutionary advantage is that sequence divergence of paralogs creates binding sites for the incorporation of additional proteins into the DMT. For example, the two trypanosomatid paralogs of CFAP67 bind different partners: CFAP67A binds DMIP20 and CFAP143, while CFAP67B binds CFAP141 and DMIP17. This contrasts with the fewer binding partners of the single CFAP67 found in green algae and mammals (6, 10).

Paralogous proteins also allow for the formation of proximal-distal asymmetry. In the context of the ODA-DC, this asymmetry is important for controlling the site of waveform initiation (tip or base) and therefore the direction of waveform propagation (38). Further investigations are required to understand why some paralogs become asymmetrically distributed while others do not. Increasing evidence suggests that differential association with the IFT machinery may play a role (38, 40).

A second mechanism of increasing DMT complexity – common to all DMTs studied to date – is the incorporation of clade-specific MIPs. Our work shows that in trypanosomatids this addition is particularly pronounced, with 27 acquired proteins. Although loss of some of these proteins impacts swimming speed, most do not, suggesting their function extends beyond conventional motility, potentially playing roles in chemotactic beat control or flagellum restructuring between life stages. This added complexity may help ensure that the flagellum is reliably assembled and robust, enhancing its stability under various conditions. Further experiments will be needed to test if MIPs contribute to flagellar resilience under mechanical, chemical, or genetic stress. A better understanding of the conditions trypanosomatids encounter in their insect vectors will be necessary to guide these investigations.

Our structural analysis of trypanosomatid doublet microtubules has revealed the specific specializations endowed by evolution on this particular class of pathogen, where the flagellum is critical to their life cycle. Through a systematic evaluation of the contribution of each structure-verified protein to flagella motility, we have demonstrated the singular importance of the inner junction to flagellar motility. Looking ahead, the application of our integrated structural and genetic approach to additional axonemal complexes is poised to provide insights into both the conserved and trypanosomatid-specific mechanisms governing flagellar beating.

## Materials and Methods

### Cell culture

Promastigote-form *Leishmania tarentolae* strain P10 cells (Jena Bioscience, #LT-101) were grown in BHI medium (HIMEDIA, #N210) containing 0.005% hemin chloride (Sigma, #3741) and 10 U/mL Penicillin-Streptomycin (Gibco, #15070063) at 26°C in the dark. Cells were maintained as static suspension cultures as described in the LEXSY expression kit manual (Jena Bioscience).

Promastigote-form *L. mexicana* Cas9 T7 (26), derived from WHO strain MNYC/BZ/62/M379, were grown at 28°C in M199 medium (Life Technologies) supplemented with 2.2 g/L NaHCO_3_, 0.005% hemin, 40 mM 4-(2-Hydroxyethyl)piperazine-1-ethanesulfonic acid (HEPES) pH 7.4, 10% fetal calf serum (FCS), 50 μg/mL Nourseothricin Sulphate and 32 μg/mL Hygromycin B.

*Crithidia fasciculata* Leger strain cells (ATCC 50083) were grown in ATCC 355 media at 25°C. Cells were grown in flasks on a rocker and passaged every 2–3 days to maintain a density between 1 × 10^6^ and 1 × 10^8^ cells/mL.

### Flagella isolation and preparation of axonemes

The method for isolating *L. tarentolae* flagella was adapted from (41). Briefly, a 10 mL exponentially growing starting culture was added to 200 mL of BHI medium and grown in the dark for 24 hr to a cell density of 1 × 10^7^ cells/mL. The cells were pelleted by centrifugation and washed with phosphate-buffered saline. All subsequent steps were performed at 4°C. After washing, the cells were resuspended in PIPES solution (10 mM piperazine-N,N′-bis(2-ethanesulfonic acid), 10 mM NaCl, 1 mM CaCl_2_, 1 mM MgCl_2_, 0.32 M sucrose, adjusted to pH 7.2). Following resuspension, CaCl_2_ was added to a final concentration of 100 mM and a ProteaseArrest (G-Biosciences) protease inhibitor cocktail was added. Flagella were then sheared from the cell bodies by drawing the cell suspension through a 20-gauge needle using a 10 mL Hamilton syringe 100 times. This process was monitored using a light microscope and continued until the *L. tarentolae* cells were immotile. The isolated flagella were separated from the cell bodies by centrifugation over a 33% sucrose cushion where the flagella remained above the cushion and the cell bodies pelleted. The flagella layer was removed and collected into a cell pellet by ultracentrifugation at 100,000 ξ *g* for 1 h at 4°C. The pellet was then resuspended in PMDEKP (10 mM PIPES, 25 mM KCl, 5 mM MgSO_4_, 0.5 mM EGTA, Protease Arrest (G-Biosciences)).

*L. tarentolae* flagella were demembranated by the addition of 1% NP-40 to the sample in fresh PMDEKP at 4°C while gently rotating for 30 min. Once the membrane had been broken, the solution was centrifuged at 10,000 ξ *g* for 10 min to separate the axoneme filaments (pellet) from the soluble proteins and detergent. After washing twice with fresh PIPES solution, the axonemes were splayed at a final concentration of 0.5 mg/mL by adding 10 mM Mg-ATP and 750 μM CaCl_2_ in HMDEKP while being rotated at room temperature for 1 h. Splayed axonemes were concentrated by centrifugation at 2,500 × *g* to an absorbance reading at 280 nm of 6.5 and immediately used to prepare cryo-EM grids.

*C. fasciculata* flagella were prepared using a mechanical approach adapted from (15) that combined flagella isolation with demembranation. To begin, 5 × 10^8^ *C. fasciculata* cells were pelleted by centrifugation at 2000 × *g* for 10 min and washed twice with buffer (30 mM HEPES pH 7.4, 30 mM KCl, 0.5 mM EGTA, 1 mM DTT, Roche Protease Inhibitor). To remove the cell membrane, 100 mL wash buffer supplemented with 2% Triton X-100 and DNase I (Roche) was added and rotated at room temperature for 1 h. The suspension was drawn through a 25-gauge syringe needle 20 times on ice to facilitate flagellar detachment and membrane breakdown. 1 mM CaCl_2_ was added to the demembranated *C. fasciculata* and rotated at 4°C for 1 h to solubilize the subpellicular microtubules. The 25-gauge syringe was applied 20 more times on ice to assist loosening of the axonemal bundles. Axonemes were then centrifuged at 3000 × *g* at 4°C for 10 min and the supernatant was removed. Axonemes were purified away from cell body debris by one further centrifugation step over a 30% sucrose cushion at 800 × *g* at 4°C for 5 min (30% w/v sucrose in wash buffer). Axonemes from 200 μL of the upper fraction of the buffer-sucrose interface were collected and washed twice in wash buffer with 0.1% Triton X-100 by centrifugation at 3000 × *g* at 4°C for 10 min. The pellet was then resuspended in wash buffer with 0.05% Triton X-100 to achieve a concentration of 10 mg/mL for cryo-EM, negative stain, or mass spectrometry preparation.

### Mass spectrometry analysis (*L*. *tarentolae*)

Mass spectrometry analysis of purified *L. tarentolae* DMTs – provided as a band excised from an SDS-PAGE gel – was conducted at the Taplin Mass Spectrometry Facility at Harvard Medical School. To begin, the band was washed and dehydrated with acetonitrile and vacuum concentration. The gel pieces were then rehydrated with 50 mM ammonium bicarbonate solution containing 12.5 ng/mL trypsin (Promega) and incubated for 45 min at 4°C. At 45 minutes, the trypsin solution was replaced with 50 mM ammonium bicarbonate solution and samples left at 37°C overnight. After the overnight incubation, the peptides were extracted using a single wash with a solution containing 50% acetonitrile and 1% formic acid. The washed sample was then dried again in a vacuum concentrator. The dried extracts were resuspended in 10 mL of solvent A (2.5% acetonitrile, 0.1% formic acid) and loaded onto a pre-equilibrated reverse-phase capillary column (100 mm inner diameter x 30 cm length) containing 2.6 mm C18 spherical silica beads using a Famos auto sampler (LC Packings). The peptides were eluted with increasing concentrations of solvent B (97.5% acetonitrile, 0.1% formic acid). At this time, the eluted peptides were subjected to electrospray ionization and entered into an LTQ Orbitrap Velos Pro ion-trap mass spectrometer (Thermo Fisher Scientific). Peptides were detected, isolated, and fragmented to produce a tandem mass spectrum for each peptide. Tandem mass spectra were acquired and analyzed using Sequest (Thermo Fisher Scientific). Peptides masses were searched against the *L. tarentolae* Parrot-TarII reference and non-reference proteomes obtained from TriTrypDB. Data were filtered to a peptide false discovery rate (FDR) of 1-2%.

### Mass spectrometry analysis (*C*. *fasciculata*)

Mass spectrometry analysis of purified *C. fasciculata* axonemes (10 mg/mL) – provided as a vitrified solution – was conducted at the Proteomics and Metabolomics Facility at the University of Nebraska-Lincoln. All MS/MS samples were analyzed using Mascot (Matrix Science, v. 2.6.1). Mascot was set up to search the *C. fasciculata* proteome obtained from TriTrypDB and a list of common contaminants (cRAP_20150130). Mascot was searched with a fragment ion mass tolerance of 0.060 Da and a parent ion tolerance of 15.0 PPM. Asparagine and glutamine deamidation, methionine oxidation and cysteine carbamidomethylation were considered as possible post-translational modifications. The mass spectrometry proteomics data have been deposited to the ProteomeXchange Consortium via the PRIDE (42) partner repository with the dataset identifier PXD057610. The annotated mass spectrometry data are provided in Data S1.

### Negative-stain electron microscopy

Negative-stain electron microscopy was used to assess sample quality. 4 µL of *L. tarentolae* purified flagella at an A280 reading of 2-3 was deposited onto glow-discharged continuous carbon support grids. The sample was incubated for 1 minute and immediately blotted and washed with distilled water. After washing, the grid was blotted once more and incubated with 4 µL of 1.5% uranyl formate solution for one minute. The grid was blotted once more to remove excess stain and air-dried at room temperature for one minute. The sample was examined with a Tecnai T12 electron microscope (Thermo Fisher Scientific) operating at 120 kV accelerating voltage with a LaB6 filament, equipped with a Gatan UltraScan 895 (4k x 4k) CCD at the Molecular Electron Microscopy suite (MEMs) at Harvard Medical School.

Similarly, 4 μL of purified *C. fasciculata* axoneme samples at a concentration of 1 mg/mL was applied onto a glow-discharged C-flat 200-mesh copper support grid (Electron Microscopy Sciences). After 1 min sample incubation, the grid was blotted with filter paper to remove excess sample, and immediately washed twice with 4 μL of 2% uranyl formate solution. The grid was then incubated with 4 μL of 2% uranyl formate for an additional 1 min, blotted with filter paper, and air-dried at room temperature. The sample was then analyzed on a JEOL JEM-1400 120 kV transmission electron microscope equipped with an AMT XR111 high-speed 4k 2k CCD camera at Washington University Center for Cellular Imaging (WUCCI), using a nominal magnification of 52,000 × at a pixel size of 2.13 Å.

### Cryo-EM grid preparation

Cryo-EM grids of *L. tarentolae* splayed axonemes were prepared using a Vitrobot Mark IV (Thermo Fisher Scientific) equilibrated to 5°C and 100% humidity. 3 μL aliquots of sample were applied to glow discharged Quantifoil holey carbon grids (R2/1, copper, 300 mesh) and blotted for 16-18 s with a blot force of 10 before being plunged frozen in liquid ethane.

Cryo-EM grids of *C. fasciculata* axonemes were prepared using a Vitrobot Mark IV equilibrated to 8°C and 95% humidity. 4 μL of *C. fasciculata* axoneme sample (10 mg/mL) was applied to glow-discharged Quantifoil holey carbon grids (R2/1, copper, 300-mesh) and blotted for 15 s with a blot force of 11 being plunged frozen in liquid ethane.

### Cryo-EM data collection

Cryo-EM images of splayed *L. tarentolae* axonemes were collected at the Harvard Cryo-EM Center for Structural Biology. Data were collected using a Titan Krios equipped with a BioQuantum K3 Imaging Filter (slit width 25 eV) and a K3 direct electron detector (Gatan) and operating at an acceleration voltage of 300 kV. Over the course of four data collections, 37,665 micrographs were recorded at a defocus range of -0.8 to -2.5 μm with a nominal magnification of 64,000×, which corresponds to a calibrated pixel size of 1.33 Å. All movies contained 54 frames with an exposure time of 5.7 s, resulting in a total dose of 62.6 electrons/Å^2^.

Cryo-EM images of *C. fasciculata* axonemes were collected using two Titan Krios microscopes at the Pacific Northwest Center for Cryo-EM (PNCC). Movies were recorded in super-resolution mode on a K3 camera with a BioQuantum energy filter at slit width of 20 eV. All movie series were recorded at a defocus range of −0.7 to −2.5 μm with a nominal magnification of 81,000×, corresponding to a calibrated pixel size of 1.061 Å or 1.0655 Å. A total dose of ∼60 electrons/Å^2^ on the specimen was fractionated into 50 movie frames. For both samples, SerialEM was used for semi-automatic image collection (43, 44).

### Cryo-EM image processing of the *L*. *tarentolae* DMT

Image processing was performed in RELION-4 (45), unless otherwise stated. First, a total of 37,655 movies were motion corrected and dose weighted. Contrast transfer function (CTF) parameters were estimated using CTFFIND 4.0 (46). Micrographs with predicted resolutions worse than 7 Å were discarded. To pick particles, a CRYOLO filament-picking model (47) was trained by manually selecting microtubules from 100 micrographs using the napari-boxmanager software. We applied the trained model to each dataset, specifying an inter-box distance of 82 Å to match the rise of the tubulin heterodimer. The selected particles were then transferred to RELION and extracted as helical segments with a diameter of 500 and a box size of 512 pixels. The particles were binned 2x to speed up computation in the early stages of processing. Across the four datasets, 7,883,514 particles were picked. Initial 3D alignments were performed with the map of the *C. reinhardtii* DMT (EMD-20631) (6) low-pass filtered to 15 Å as a reference. The translational sampling range was increased to 45 Å to ensure accurate 4 nm alignments. The accuracy of initial alignments was validated by examining the CFAP20/PACRG ladder in the inner junction (IJ). To further filter poorly aligned or damaged particles, we performed a 3D classification using a cylindrical mask covering the IJ. Only the best resolved classes were selected, totaling 2,008,749 particles. Once identified, these final particles were subjected to 3D classification in order to determine the periodicities needed to resolve the *L. tarentolae* DMT architecture, as detailed in (16).

To resolve the 48 nm-internal MIP architecture of the *L. tarentolae* DMT, two successive rounds of 3D classification were performed to increase particle periodicities in a stepwise fashion. First, 16-nm features of the DMT were resolved using classification with a cylindrical mask covering the repeating MIPs at the IJ, resulting in two well-resolved classes offset by 16 nm. The particles from both classes were then superimposed, had duplicates removed, and subjected to a 3D refinement to obtain a 16-nm map. Subsequently, 48-nm particles were identified by performing classification using a cylindrical mask covering the repeating MIPs at the seam of the A tubule. This classification yielded three well-resolved classes. Particles from these classes were then superimposed, had duplicates removed, and refined to yield a 48-nm map with 533,420 total particles at a resolution of 3.3 Å. 96-nm particles were obtained by classification using a cylindrical mask on the MAP densities on the A02-A03 protofilament, yielding two classes that were refined separately to return two maps, each containing half of the 96-nm repeat reporting resolutions of 3.7 Å.

To improve the quality of the 48-nm repeat map, local refinements were performed in RELION 5.0beta using Blush regularization (48). The first set of local refinements used 30 masks that focused on overlapping 16 nm longitudinal sections of 2-3 protofilaments, which improved local resolutions to 2.8-3.3 Å. We created six additional masks surrounding the B-tubule MIPs to improve these regions. B-tubule MIP refinements were performed using particle re-centering, 3D classification, and mask-focused local refinement to produce local resolutions of ∼3.8 Å. Maps were sharpened using postprocessing in RELION and DeepEMhancer (49). Local, sharpened maps were then fitted to the 48-nm consensus map and merged into a single composite map using the “vop maximum” command in ChimeraX (50). Both the RELION and DeepEMhancer maps were used for model building, while only the RELION map was used for model refinement. The data processing scheme is illustrated in fig. S1.

To identify particles containing the ODA-DC, 3D classification using a cylindrical mask positioned over protofilaments A07-A08 was applied to 48-nm particles (fig. S1). This classification yielded one class with 107,126 particles with well-resolved densities for two docking complexes separated by 24 nm. These particles were refined to obtain a map of the ODA-DC attached to the DMT surface. The quality of the density was improved further by subtracting and re-centering the complex in a smaller box (300 Å) and performing mask-focused local refinement to exclude tubulin density, which otherwise tended to dominate alignments and result in minimal improvements in the ODA-DC map quality. After local refinement, the two docking complexes of the 48-nm repeat reported resolutions ∼3.4 Å, sufficient for model building and unambiguous protein identification. The ODA-DC maps were incorporated into the final composite map using the “vop maximum” approach described above.

### Cryo-EM image processing of the *C*. *fasciculata* DMT

Three cryo-EM datasets were collected on two different microscopes at the Pacific Northwest Center for Cryo-EM (PNCC). All datasets were processed using identical procedures. Movie frames were drift-corrected and dose weighted using ‘*Patch Motion Correction*’ in cryoSPARC (51). CTF parameters were estimated using ‘*Patch CTF Estimation’* in cryoSPARC. DMTs were automatically picked from a total of 5,837 micrographs using *‘Filament Tracer’* in CryoSPARC. DMT particles were then extracted from the motion-corrected micrographs (Fourier binning from 640 pixels to 256 pixels). The step size between adjacent particle boxes was set to 82.5 Å, equivalent to the length of an α/β-tubulin heterodimer. Two rounds of 2D classifications were used to discard junk particles and off-centered DMT particles. The remaining particles underwent *‘Heterogeneous Refinement*’ using two reference models corresponding to doublet and singlet microtubules. Particles from the class of doublet were selected and exported to FREALIGN v9.11 for further local refinement. In this step we used customized scripts to minimize the alignment errors based on the geometric relationship of neighboring DMT particles (52). The refined particle set was then imported back into cryoSPARC and subjected to local refinement.

To identify particles with 48-nm periodicity (48-nm particles), we performed tubulin signal subtraction followed by 3D classification in RELION 3.1 (53) using a soft-edged cylindrical mask covering MIPs bound to protofilaments A08-A12 (red dashed circle in fig. S2). A similar strategy was used to further separate the 48-nm particles into 96-nm particles, using a soft-edged cylindrical mask covering external axonemal complexes bound to protofilaments A01-A03. The coordinates of the 48-nm particles were imported back to cryoSPARC, and the set of 48-nm particles were expanded using *‘Symmetry Expansion’* in CryoSPARC (rise 495.12 Å, twist -0.3°, helical symmetry order 3) to add neighboring particles. Duplicate particles were removed, and the expanded 48-nm particles were re-extracted with a 512-pixel box size (using Fourier binning from 640 pixels). At this step, particle datasets from two different microscopes with slightly different pixel sizes were scaled via Fourier cropping to the same pixel size (1.3319 Å) and merged for subsequent processing. A total of 241,951 48-nm particles were subjected to local refinement, local CTF refinement, and another round of local refinement, producing a final consensus 48-nm map at 3.1 Å resolution.

To improve the local resolution, we employed a similar focused refinement strategy using a set of cylindrical masks (fig. S2) as described for the *L. tarentolae* DMT but using cryoSPARC. The 36 local refined maps, with resolutions ranging from 2.7 Å to 3.1 Å, were stitched to produce the final composite map.

### Model building and protein identification

Proteins were identified directly form the *L. tarentolae* maps using a combination of previous DMT structures, AI-guided structure predictions from AlphaFold2 (AF2) (54), automated *de novo* modeling using ModelAngelo (18), and manual *de novo* modeling using Coot (55).

To begin, we compiled an AF2 library of each of the ∼500 proteins identified in our *L. tarentolae* mass spectrometry experiment (Data S1) to serve as a database of potential DMT components. We then modeled the tubulin component by rigid-body fitting α-tubulin and β-tubulin models from our AF2 database into the protofilament density. N and C-terminal extensions that did not fit into our density and reported low confidence values in AF2 were removed. To identify DMT-associated proteins, we applied ModelAngelo in ‘*build_no_seq*’ mode to each of our focused refinement maps. This generated ModelAngelo traces containing α-carbon backbones with amino acid predictions and hidden Markov model (HMM) profiles for each chain. Unknown densities were identified by searching the sequence of each chain against the *L. tarentolae* Parrot-TarII reference and non-reference proteomes using the BLAST tool in TriTrypDB (56). Hits were cross-referenced with our mass spectrometry library to confirm their presence in our cryo-EM sample. If the hits were present, the AF2 model was compared to the density of interest. If the prediction was a reasonable fit to the map, the AF2 model was used as the starting model for iterative real-space refinement in Coot. This strategy was most effective for globular proteins. For proteins with low confidence AF2 models, the ModelAngelo traces were used as starting points for manual building in Coot. For lower quality regions that resulted in a ModelAngelo trace but poor side-chain outputs, the HMM profiles were used in HMMER searches (57) against the *L. tarentolae* proteome to identify proteins within densities of interest. In some cases, it was necessary to manually merge or extend ModelAngelo chains to create starting model. After proteins were identified and their starting models built, loops or elongated N or C-termini were built manually in Coot.

### Model refinement

For each individual chain, real-space refinement was performed in Coot with torsion, planar peptide, trans peptide and Ramachandran restraints. Once all chains had been built, they were combined into a single PDB file and refined against the composite map using Phenix real-space refinement (58). During refinement, secondary structure, Ramachandran, and rotamer restraints were applied with the weighting of nonbonded restraints set to 500. After 5 macrocycles, the model was corrected in Coot, especially focusing on removing clashes between MIPs and tubulin. Finally, our model was completed by running Phenix real-space refinement with five macro cycles of global minimization. The quality of the refined model was analyzed by MolProbity (59) with statistics reported in Table S1.

### Conservation Analysis

To analyze the evolutionary conservation of the identified proteins, we sampled protein sequences from 26 different eukaryotic species with and without flagella. We first constructed a phylogenetic tree using the interactive tree of life (ITOL) (60). Individual branches were confirmed using PhyloT (https://phylot.biobyte.de/). Sequences for each *L. tarentolae* protein identified in this study were obtained from TriTryp. To identify homologs, we performed a Position-Specific Iterated BLAST (PSI-BLAST) (61) across non-redundant protein sequence databases of the respective eukaryotic species. Hits of similar length to the query sequence with an e-value below 0.005 were considered potential homologs. We further confirmed putative homologs by comparing their predicted AF2 structures to those determined in this study. Paralogs were identified as multiple confirmed homologous hits from the same species. Additionally, we conducted a second PSI-BLAST search using *C. reinhardtii* MIP sequences to validate conserved homologs identified in previous work (6).

### DMIP1 resequencing

During model building, we realized that the annotated sequence of DMIP1 in *L. tarentolae* (LtaP24.2160) was shorter than in *C. fasciculata* or in the closely related *L. mexicana* species. Analysis of the gene model suggested a frameshift error introduced during sequencing. To verify the error, we designed forward (5´-GCATACAGTGCAAGCCCATAAGCGC-3´) and reverse (5´-CGTCCGCATGTGCTCACAGAGCGC-3´) primers and amplified the DMIP1 coding region from genomic DNA isolated from *L. tarentolae* laboratory strain P10 using Phusion high-fidelity DNA polymerase (Thermo Fisher, #F631S). The PCR product was gel purified and sequenced with long-read sequencing (Plasmidsaurus). The corrected gene model, which is exactly the same length (266 residues) as in *L. mexicana* and 93% identical, was used to build the atomic model of DMIP1.

### CRISPR-Cas9 gene knockouts

Gene deletions in *L. mexicana* were done as described in (26, 62). Primer sequences were obtained from www.LeishGEdit.net using the latest LeishGEdit v4.2 barcoded primer design (27). Knockouts were generated from populations transfected with two sgRNA templates and two donor DNA fragments carrying drug resistance markers, amplified from pT plasmids (26) using 96-well plate formats (62). Gene deletion mutants were selected in M199 medium containing 5 μg/mL Blasticidin and 20 μg/mL puromycin dihydrochloride.

### Knockout verification

Genomic DNA of drug-selected mutants was isolated using the protocol from (63). Extracted genomic DNA was subjected to diagnostic PCRs to test for the presence of the target gene open reading frame (ORF) in putative knockout lines and the parental cell line as previously shown in (1, 26) and using primers designed by Primer3 (64) as published in (1). As a control, to test for presence of genomic DNA, a second PCR reaction was performed to amplify the ORF of the phosphomannomutase gene (LmxM.36.1960).

### Motility assays

Swimming behavior was measured from cultures with a cell density of approximately 6x10^6^ cells/mL. Three samples were measured for each cell line. For each sample, 5 μL of cell culture was placed on a glass slide in a 250-μm deep chamber covered with a 1.5 mm cover slip and imaged using darkfield illumination with a 10× NA 0.3 objective on a Axio Imager.Z2 microscope (Zeiss) at the ambient temperature of 25–28°C. Movies were recorded on an ORCA-Flash4.0 camera (Hamamatsu Photonics) at a frame rate of 5 Hz and. Swimming trajectories of parental and mutant cell lines were tracked using the method described in (28) with the modifications reported in (1). For each cell, we measured mean speed and swimming path directionality (defined as the mean velocity as a fraction of mean speed, equivalent to the ratio of displacement achieved to the distance travelled to reach that point). Data were plotted using Prism v10 (GraphPad). Statistical significance was determined using one-way ANOVA with Dunnett’s multiple comparisons test.

### Distance measurements

To measure the distance from the kinetoplast to the start of the fluorescence signal, we first manually evaluated the data available for each tagged cell line in the *T. brucei* TrypTag dataset (23). We excluded cell lines where a large proportion of the population had very low signal intensity or where there was high cytoplasmic signal background, and supplemented the list with controls: p197 (Tb927.10.15750) and p166 (Tb927.11.3290) as tripartite attachment complex markers (65, 66), SAS6 (Tb927.9.10550) and POC5 (Tb927.10.7600) as basal body markers (67, 68), TZP150 (Tb927.7.2150) as a transition zone marker (69), Basalin (Tb927.7.3130) as a basal plate marker (69), and RSP9 (Tb927.8.810), PF16 (Tb927.1.2670), OADα (Tb927.3.930) and DCR2 (Tb927.11.7240) as axoneme markers. Using the TrypTag Python module [https://guthub.com/zephyris/tryptag], we automatically retrieved TrypTag data and selected only 1K1N cells for analysis. For each cell, maximal signal intensity in the mNG channel was measured in circles radius *x* up to 25 px centered on the centroid of the kinetoplast DNA stain signal. This gives a monotonic curve suitable for measuring both point-like (e.g. transition zone) and the start of linear (e.g. axoneme) structures. For each cell, this was fitted to a sigmoid curve: x=L1+e−k(x−x0). We took values of *x*_*0*_ from good fits (*R*^2^>0.95) with a sigmoid midpoint within the data bounds (*0*<*x*_*0*_<^2^5), a sharp slope (*k*>0.3, based on typical image sharpness due to the point spread function) and good signal intensity (*L*> 1000, selected based on typical signal intensity in images). Outliers with *x*_0_, *k* or *L* more than two standard deviations from the cell line mean were excluded, and remaining *x*_*0*_ values taken as measures of distance from the centroid of the kinetoplast to the start of the mNG signal. This measurement strategy is not suitable for measuring distance to the start of the distal ODA-DC-associated protein signals as, on this length scale, the nearest bright signal to the kinetoplast may well not be the same cell and does not account for flagellum bending. Instead, these were measured manually in ImageJ (70) by tracing from the center of the kinetoplast of 1K1N cells, along the flagellum as seen in the phase contrast image, to the point of estimated 50% maximal signal intensity in the mNG channel.

### CRISPR-Cas9 gene tagging

Genes were tagged with mNeonGreen (mNG)(71) in their endogenous locus in *L. mexicana* Cas9 T7 as described in (26, 62). Primer sequences (provided in Table S2) were obtained from www.LeishGEdit.net (27) to generate the sgRNA template fragment and to amplify the donor DNA fragment from pPLOT-mNG-Blast (26). Transfected cells were selected in M199 medium containing 5 μg/mL Blasticidin for two culture passages before removing drug selection.

### Imaging of tagged *L*. *mexicana*

The mNeonGreen-tagged proteins were imaged in live cells stained with 10 µg/mL Hoechst 33342 as described in (37) using a Nikon Ti2 microscope 100x numerical aperture (NA) 1.4 oil immersion objective, and images were captured with a Hamamatsu Orca Flash 4.0 camera.

## Supplementary Material

Supplementary Materials

## Data Availability

The composite cryo-EM map of the 48-nm repeat unit of DMTs from *L. tarentolae* flagella has been deposited to the Electron Microscopy Data Bank (EMDB; https://www.ebi.ac.uk/pdbe/emdb/) with the accession code EMD-47661. The atomic model of the *L. tarentolae* DMT has been deposited in the Protein Data Bank (PDB; https://www.rcsb.org/) with accession code 9E78. Each focused refinement used to generate the composite was also deposited in the EMDB with the accession codes: EMD-47607-EMD-47610, EMD-47612-EMD-47620, EMD-47622, EMD-47624, EMD-47627, EMD-47630, EMD-47633, EMD-47635, EMD-47636, EMD-47638-EMD-47651. The composite cryo-EM map of the 48-nm repeat unit of DMTs from *C. fasciculata* flagella has been deposited with the accession code EMD-47684. Mask-focused refinements of the *C. fasciculata ponticuli* and ODA-DC have been deposited with accession codes EMD-47680 to EMD-47683. The genomic sequence of *L. tarentolae* DMIP1 has been deposited to GenBank with accession number PQ497540. Mass spectrometry data of *C. fasciculata* axonemes have been deposited to ProteomeXchange with identifier PXD057610.

## Synopsis

Structural analysis of native doublet microtubules from two trypanosomatid species defines the species-specific adaptations of a ciliated parasite.
