# Supplementary material for: Evolutionary adaptations of doublet microtubules in trypanosomatid parasites
Source: Science. Author manuscript; Available in PMC 2025 Jul 28. (PMC7617938; doi:10.1126/science.adr5507)
Supplement: Supplementary Materials [file EMS204082-supplement-Supplementary_Materials.pdf]

List of Supplementary Materials

Figs. S1 to S23

Tables S1 to S5

Science
AAAS

Supplementary Materials for

Evolutionary adaptations of doublet microtubules in trypanosomatid parasites

Matthew H. Doran, Qingwei Niu, Jianwei Zeng, Tom Beneke, James Smith, Peter Ren, Sophia Fochler, Adrian Coscia, Johanna L. Höög, Shimi Meleppattu, Polina V. Lishko, Richard J. Wheeler, Eva Gluenz, Rui Zhang, Alan Brown

Corresponding authors: zhangrui@wustl.edu and alan_brown@hms.harvard.edu

The PDF file includes:

Figs. S1 to S23
Tables S1 to S2

Other Supplementary Materials for this manuscript include the following:

Data S1 to S3

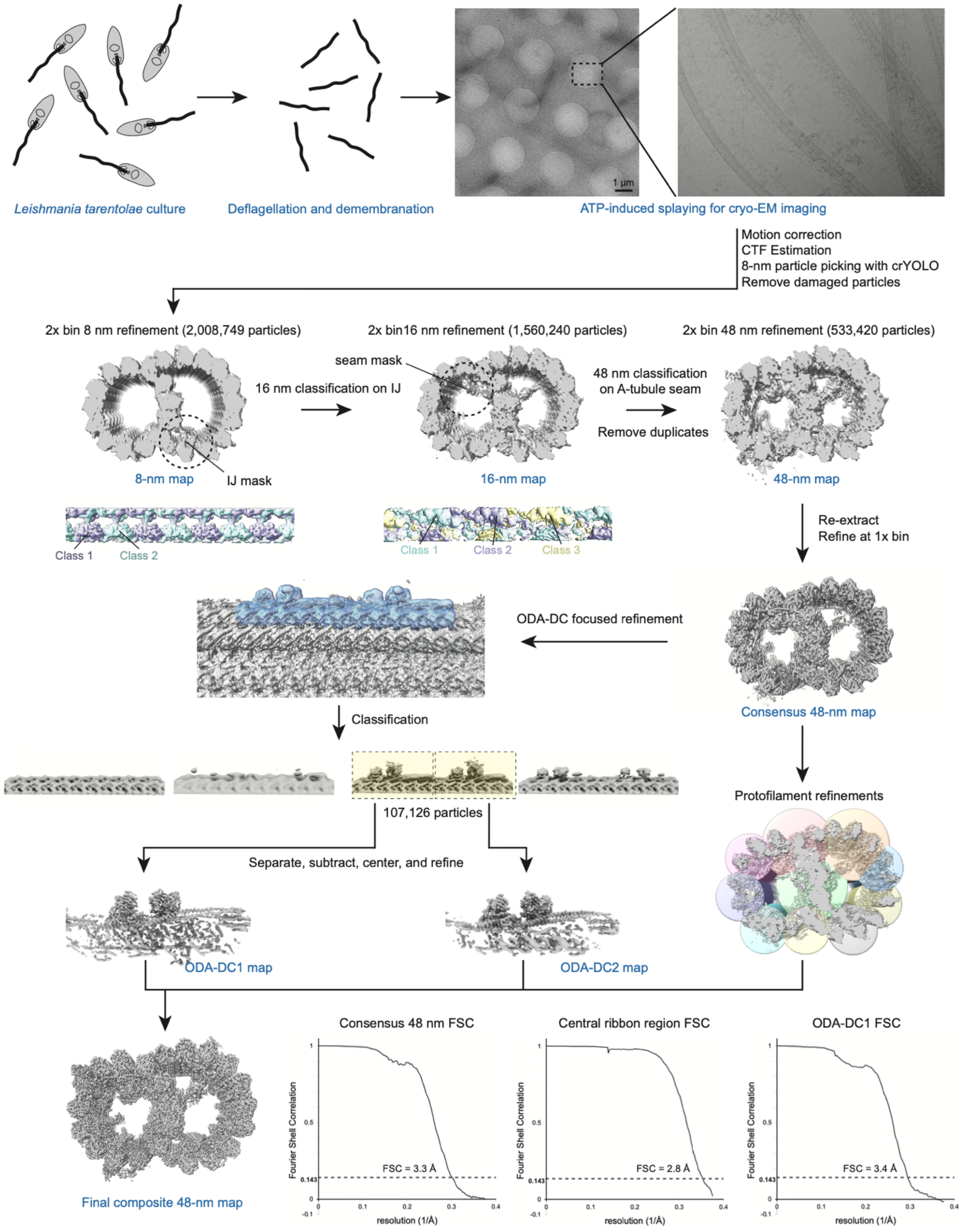

Figure S1. Cryo-EM processing of the *L. tarentolae* DMT. Schematic of the processing pipeline from preparation of doublet microtubules to formation of the composite map. Fourier shell correlation (FSC) curves are shown for the consensus refinement, a focused refinement, and the ODA-DC.

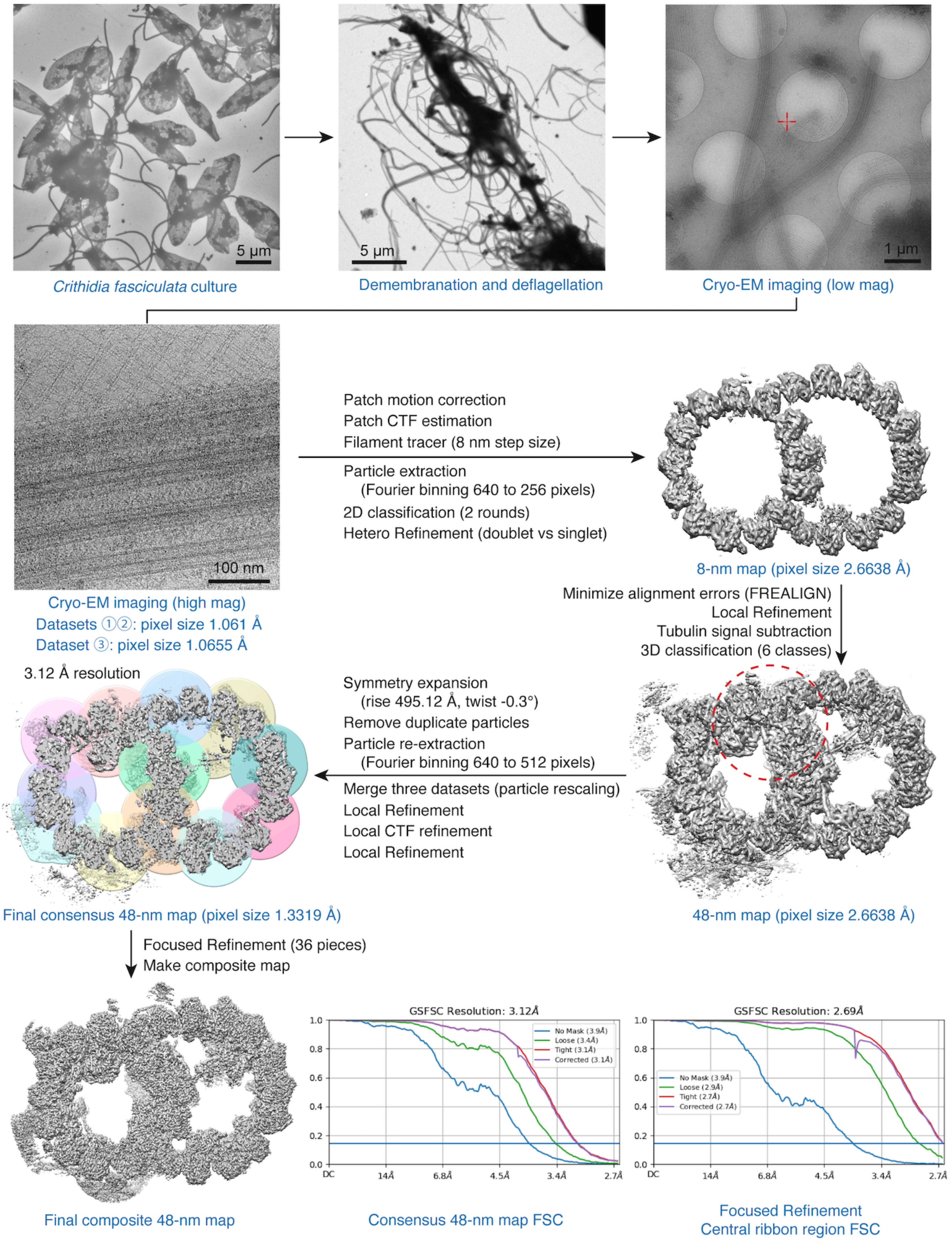

Figure S2. Cryo-EM processing of the *C. fasciculata* DMT. Schematic of the processing pipeline from preparation of doublet microtubules to formation of the composite map. Fourier shell correlation (FSC) curves are shown for the consensus refinement and a focused refinement.

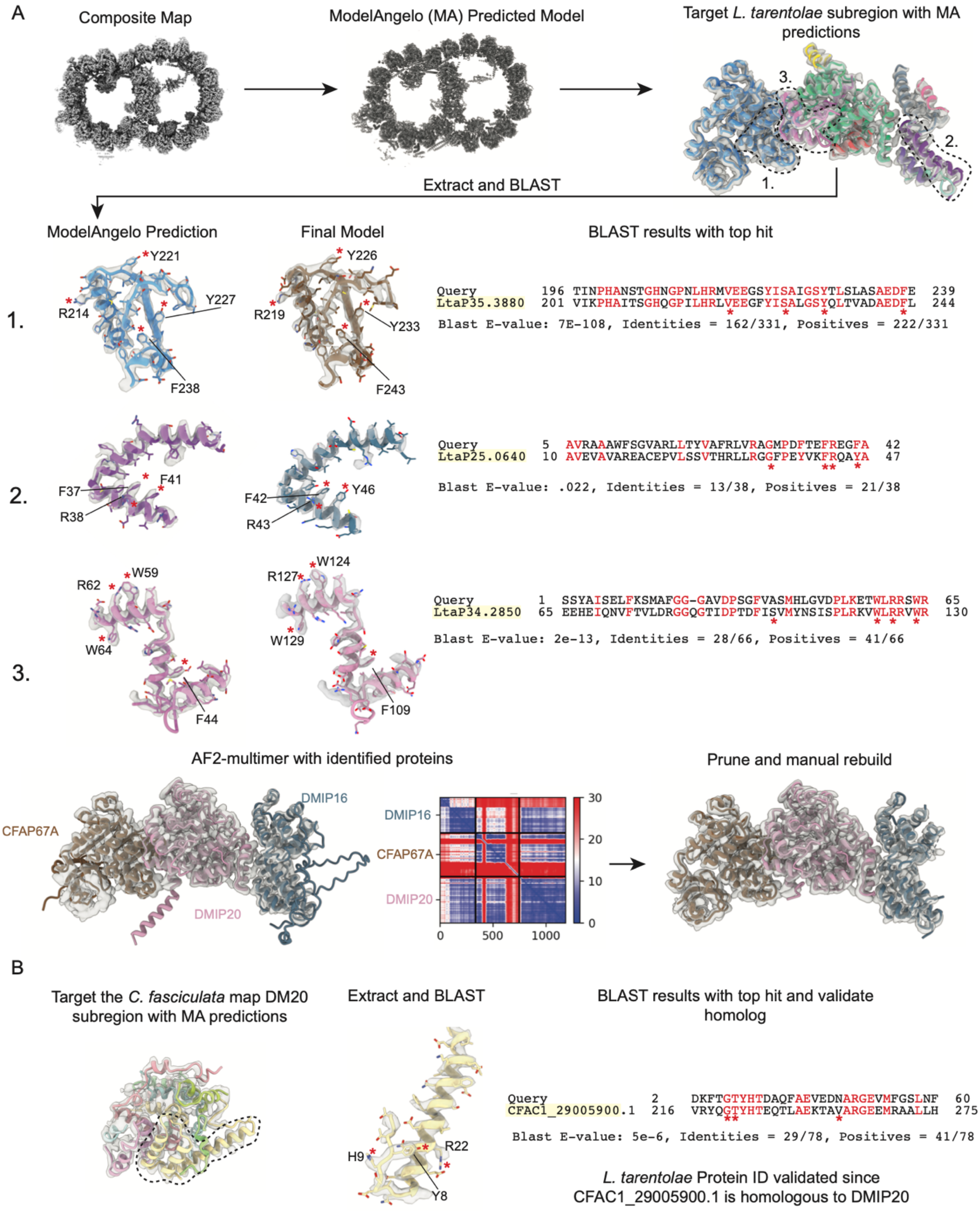

Figure S3. Protein identification strategies. (**A**) An example showing how ModelAngelo was used to identify proteins (in this case CFAP67A, DIMP20, DMIP16) in the *L. tarentolae* DMT cryo-EM map. Sequences were extracted from the ModelAngelo model and BLAST against the *L. tarentolae* proteome. The alignments show the query sequence from ModelAngelo and top hit identified by BLAST, with E-values below. Visual inspection of key residues (asterisks) was used to confirm a correct solution had been identified. AlphaFold2 (AF2) was then used to model individual proteins or complexes that were then fitted back into the density, replacing the ModelAngelo model. The AF2 models were then pruned and manually rebuilt if needed. (**B**) Example (using DMIP20) of how the *C. fasciculata* map was used to independently validate protein identification.

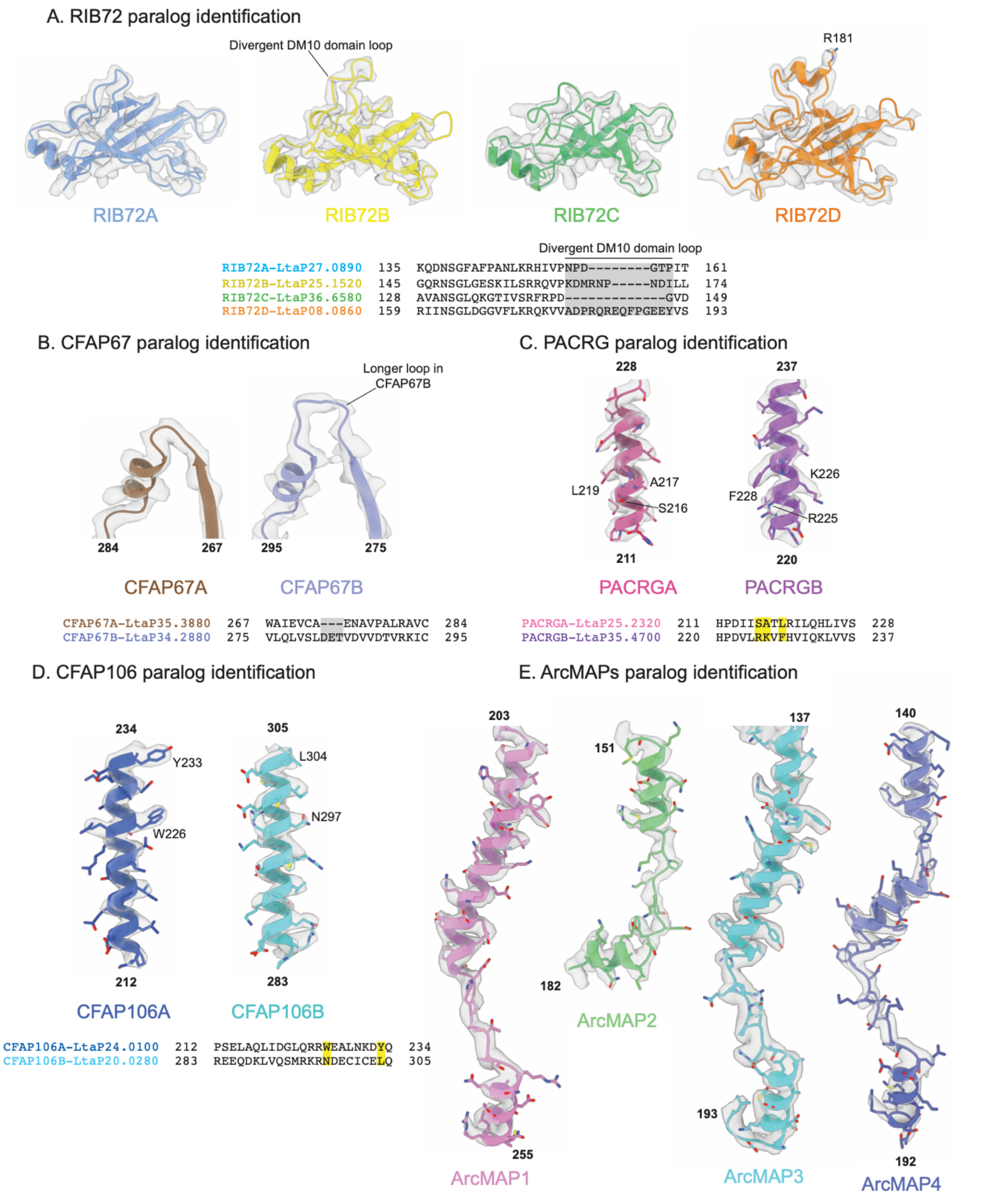

Figure S4. Paralog identification. (**A**) RIB72 paralogs can be distinguished by the length of a divergent loop in a DM10 domain. (**B**) Example of how loop length can distinguish between CFAP67 paralogs. (**C**) Example of sidechain density used to distinguish between PACRG paralogs. (**D**) Example of sidechain density used to distinguish between CFAP106 paralogs. (**E**) ArcMAP paralogs can distinguished based on helix length and sidechain differences.

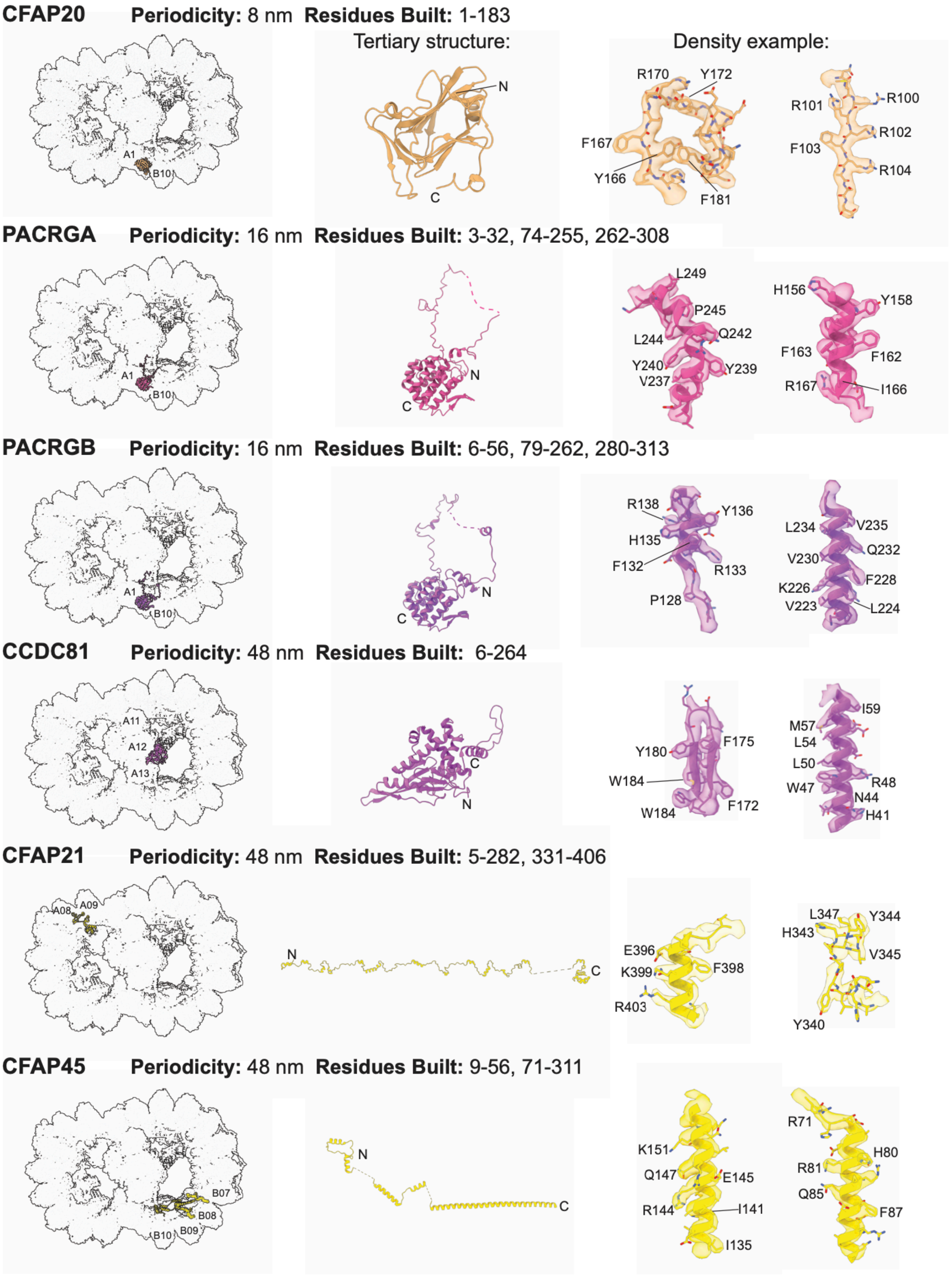

Figure S5. Location, tertiary structure, and density examples for conserved microtubule inner proteins CFAP20, PACRG, CCDC81, CFAP21 and CFAP45.

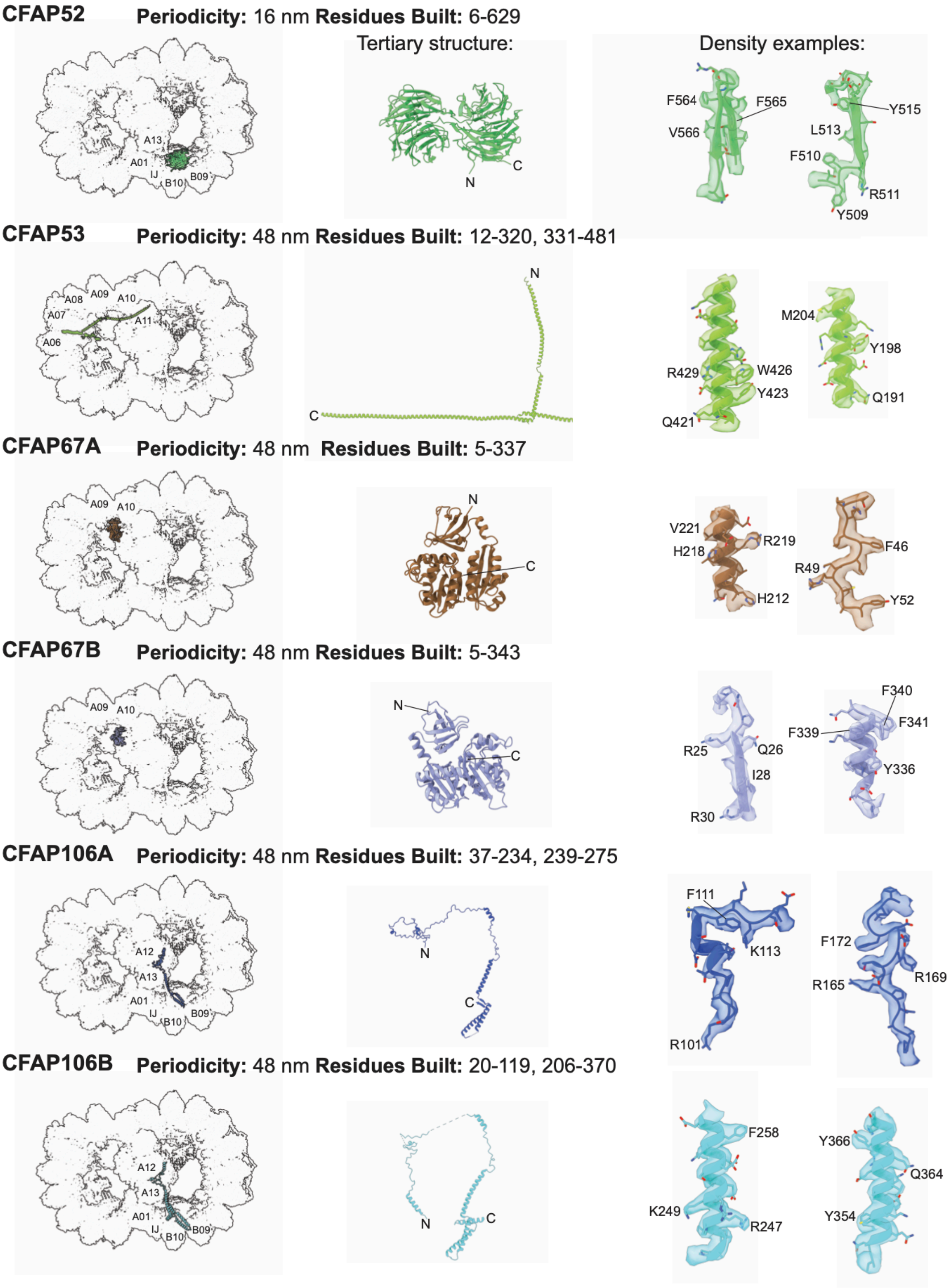

Figure S6. Location, tertiary structure, and density examples for conserved microtubule inner proteins CFAP52, CFAP53, CFAP67A/B and CFAP106A/B.

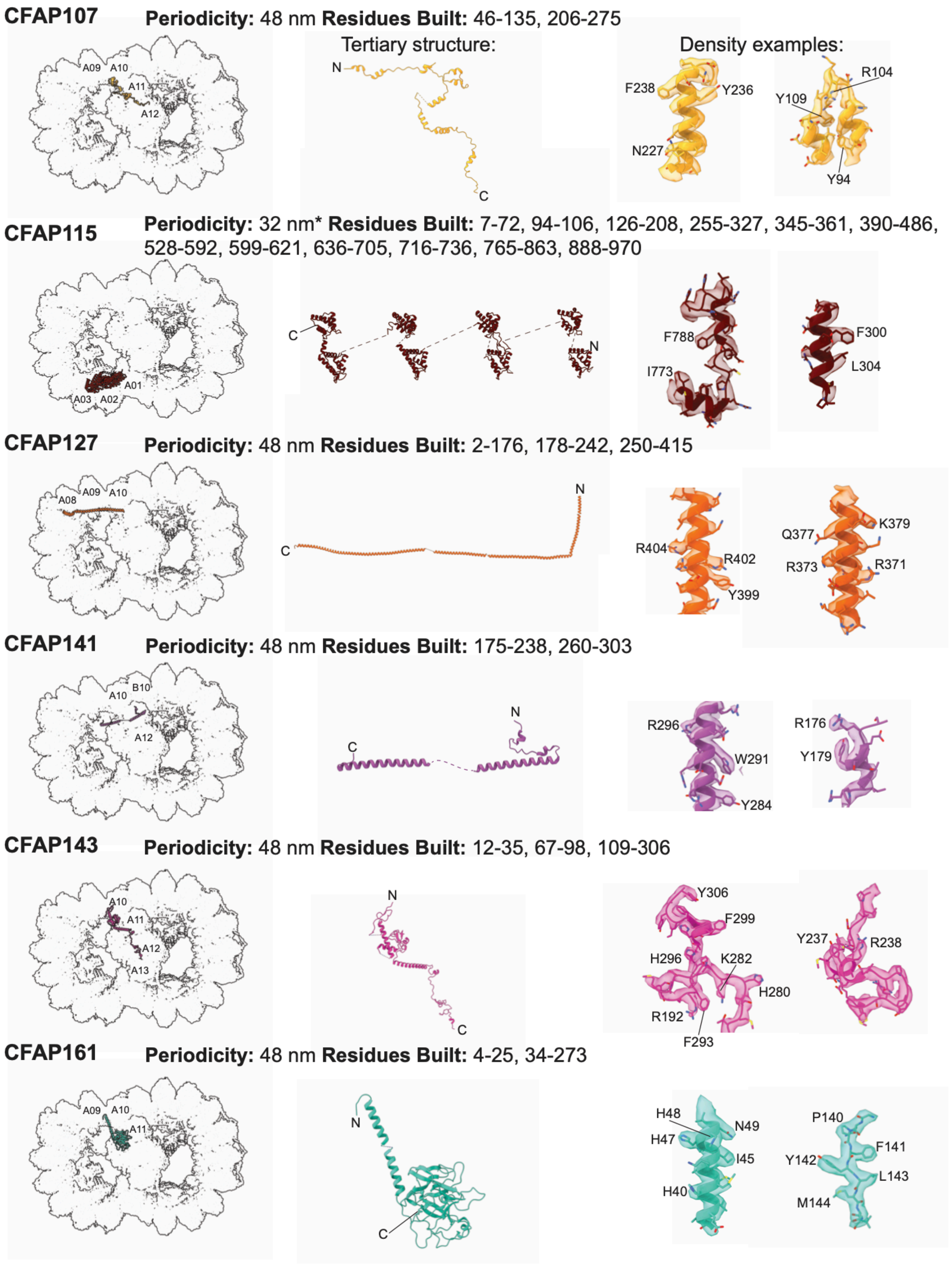

Figure S7. Location, tertiary structure, and density examples for conserved microtubule inner proteins CFAP107, CFAP115, CFAP127, CFAP141, CFAP143 and CFAP161.

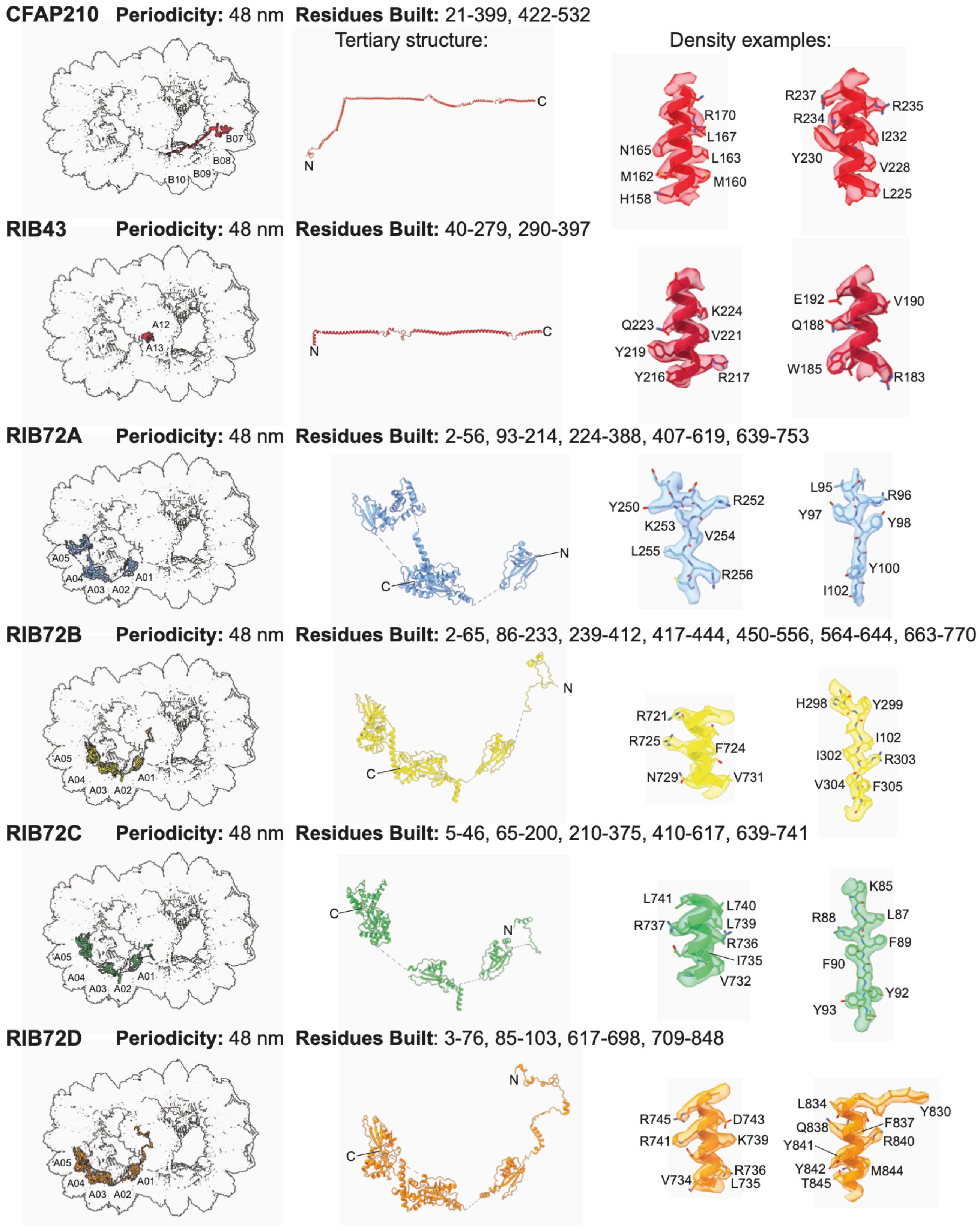

Figure S8. Location, tertiary structure, and density examples for conserved microtubule inner proteins CFAP210, RIB43 and RIB72A-D.

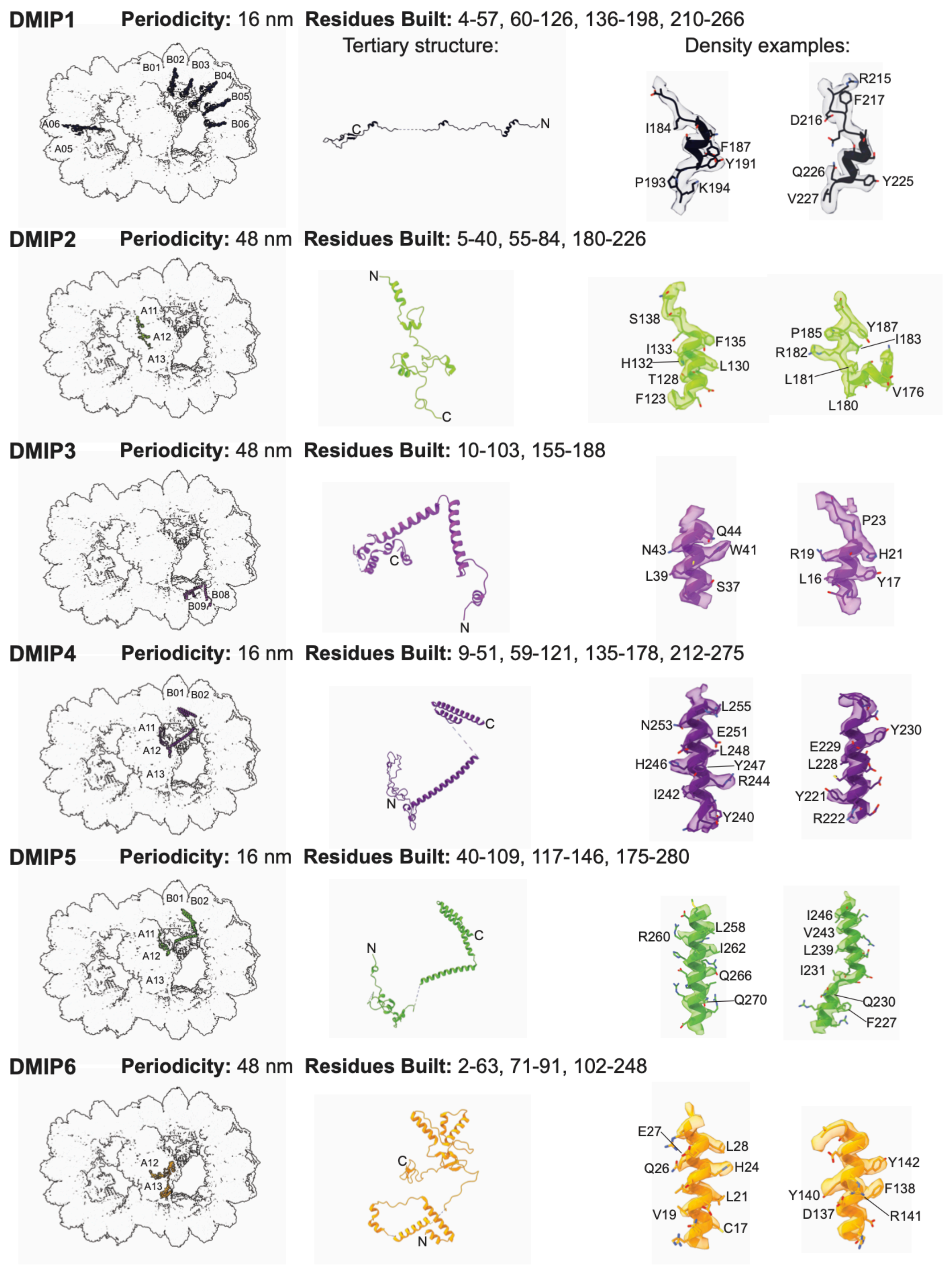

Figure S9. Location, tertiary structure, and density examples for doublet microtubule inner protein (DMIP) 1-6.

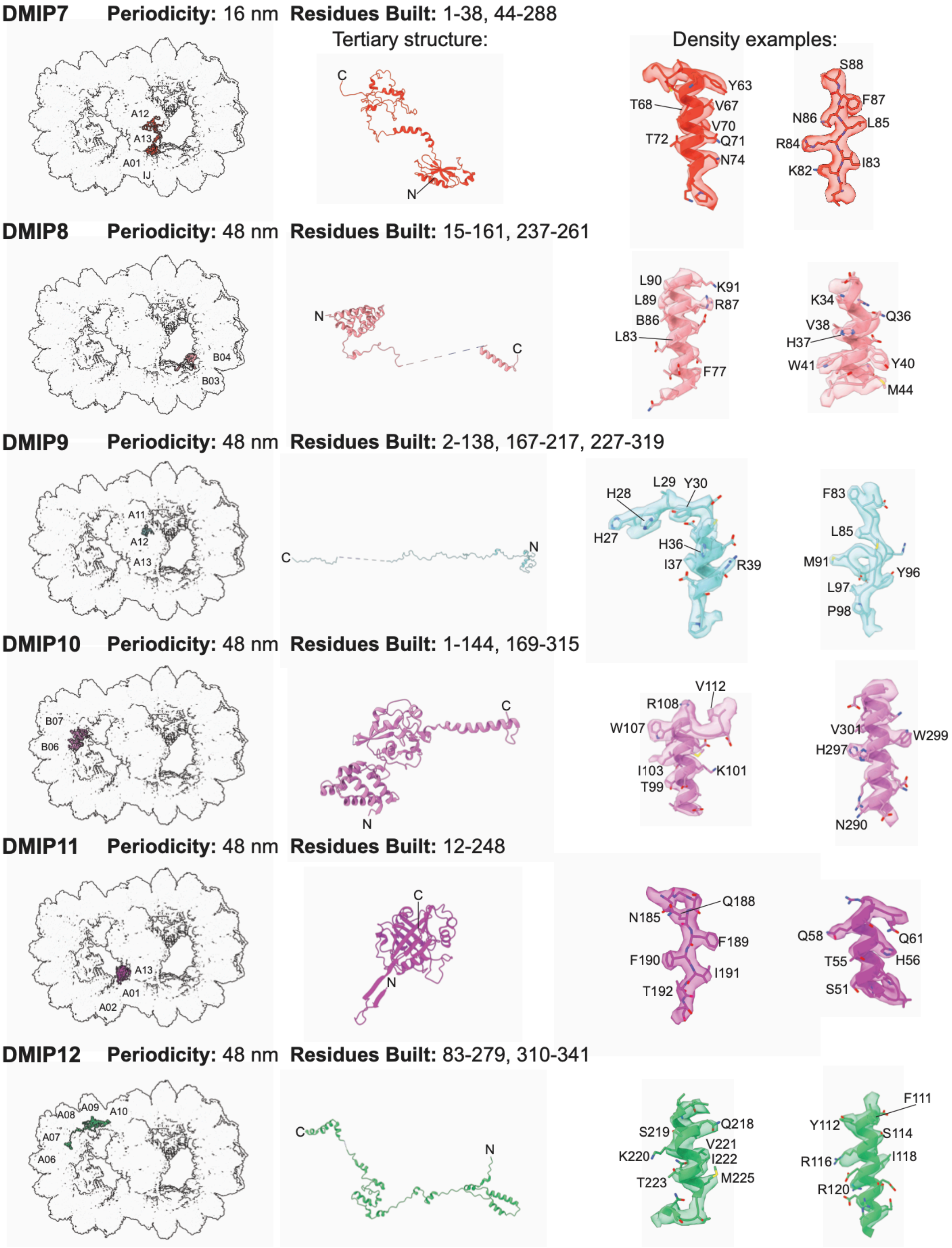

Figure S10. Location, tertiary structure, and density examples for doublet microtubule inner protein (DMIP) 7-12.

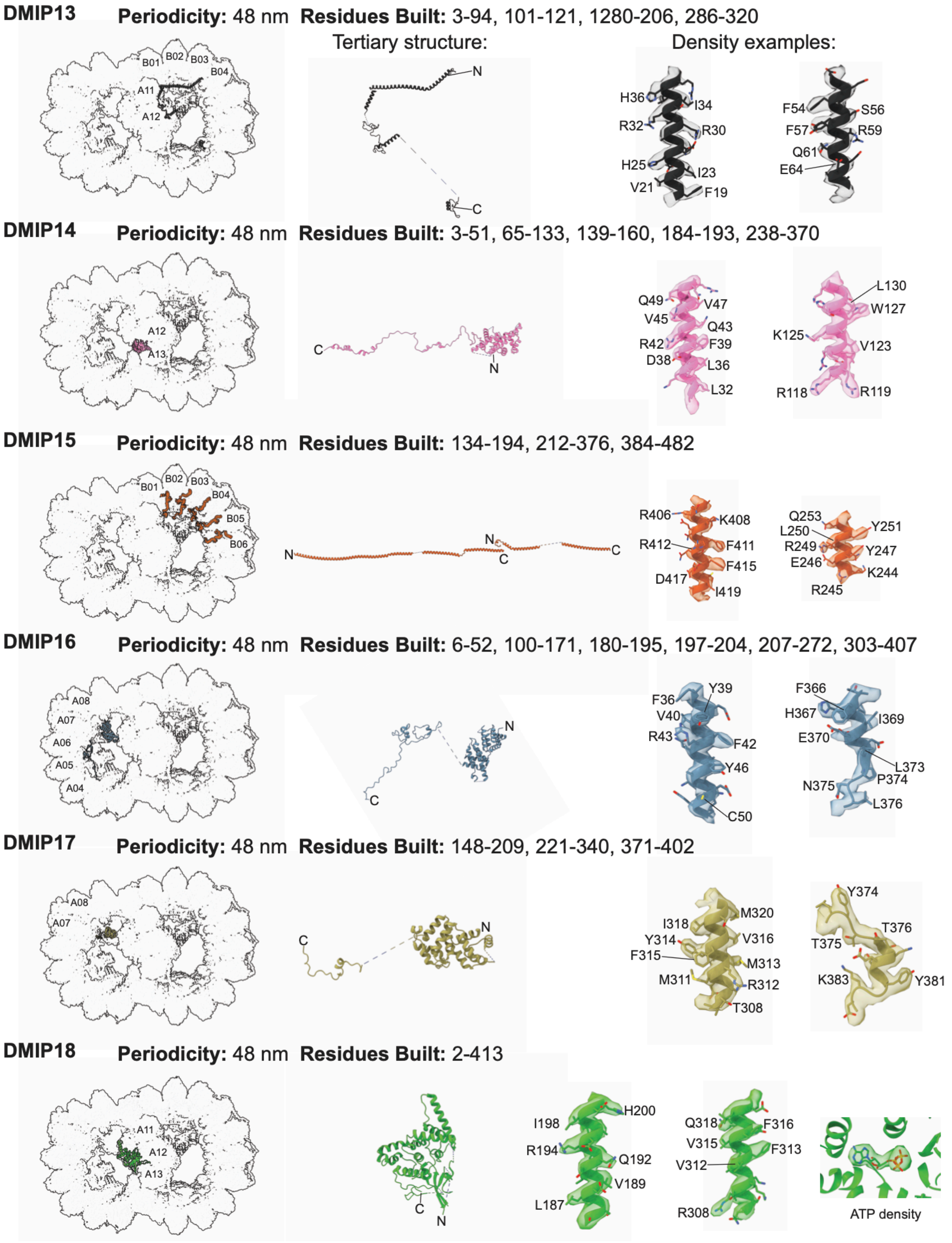

Figure S11. Location, tertiary structure, and density examples for doublet microtubule inner protein (DMIP) 13-18.

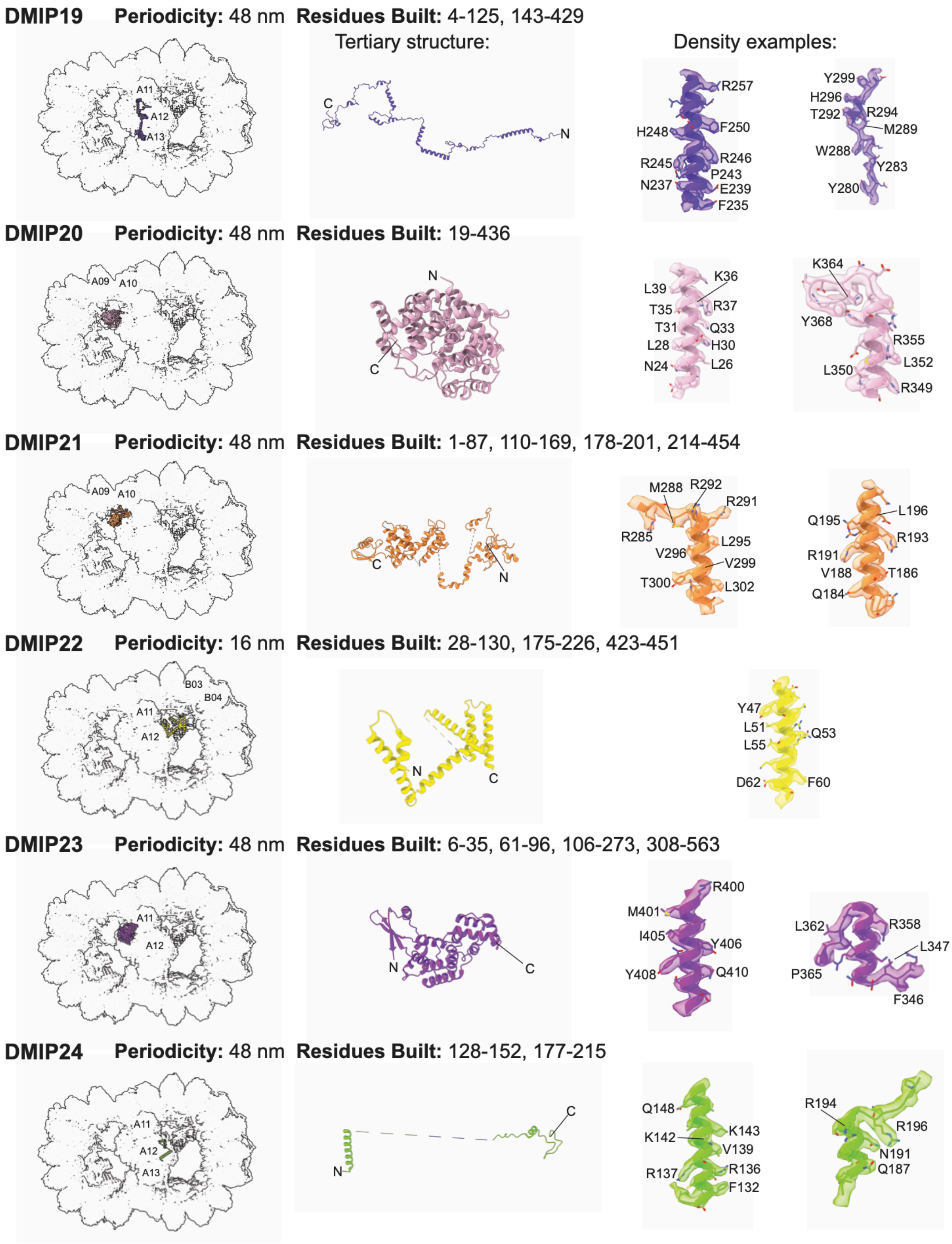

Figure S12. Location, tertiary structure, and density examples for doublet microtubule inner protein (DMIP) 19-24.

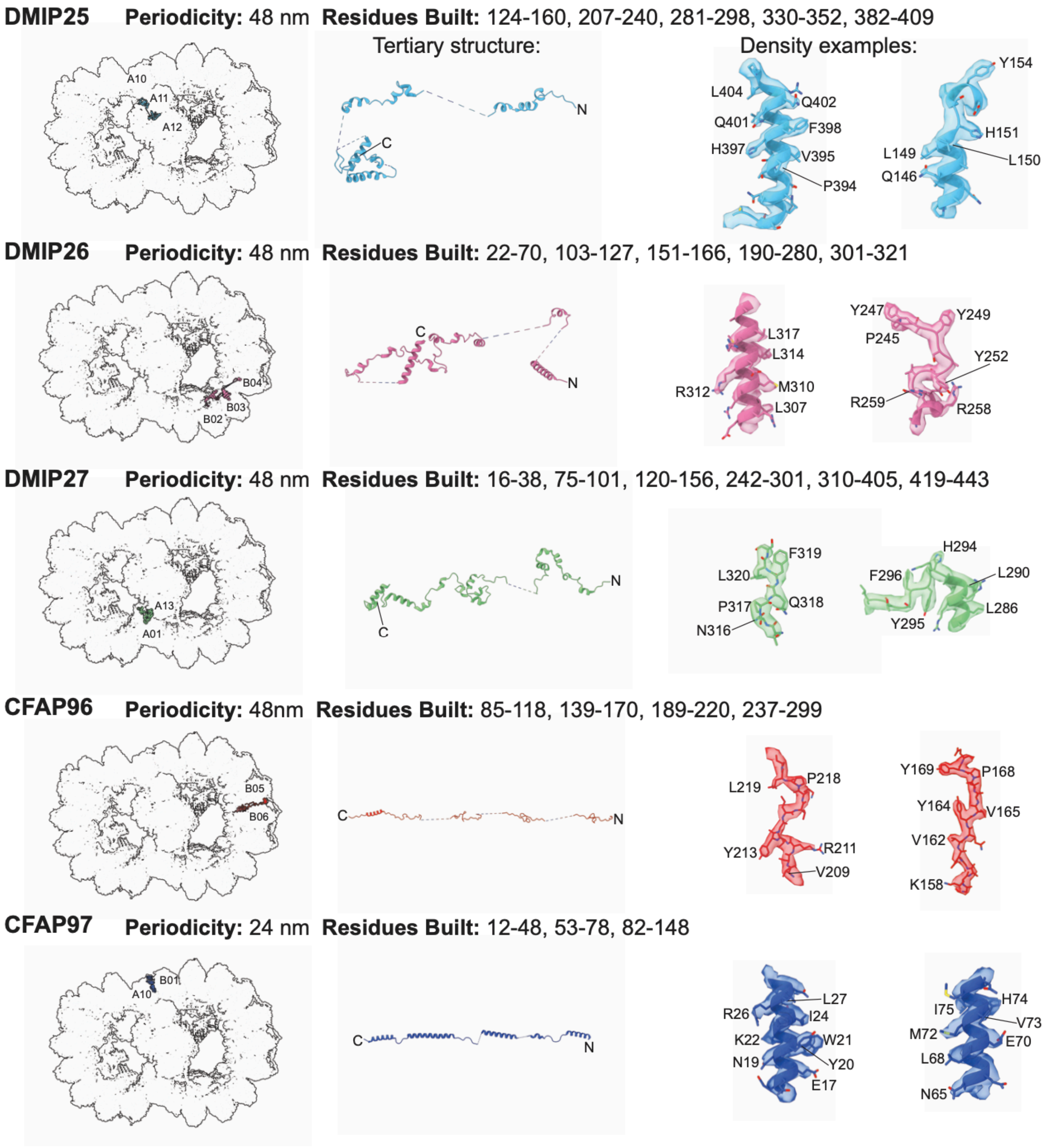

Figure S13. Location, tertiary structure, and density examples for doublet microtubule inner protein (DMIP) 25-27 and doublet microtubule-associated proteins CFAP96 and CFAP97.

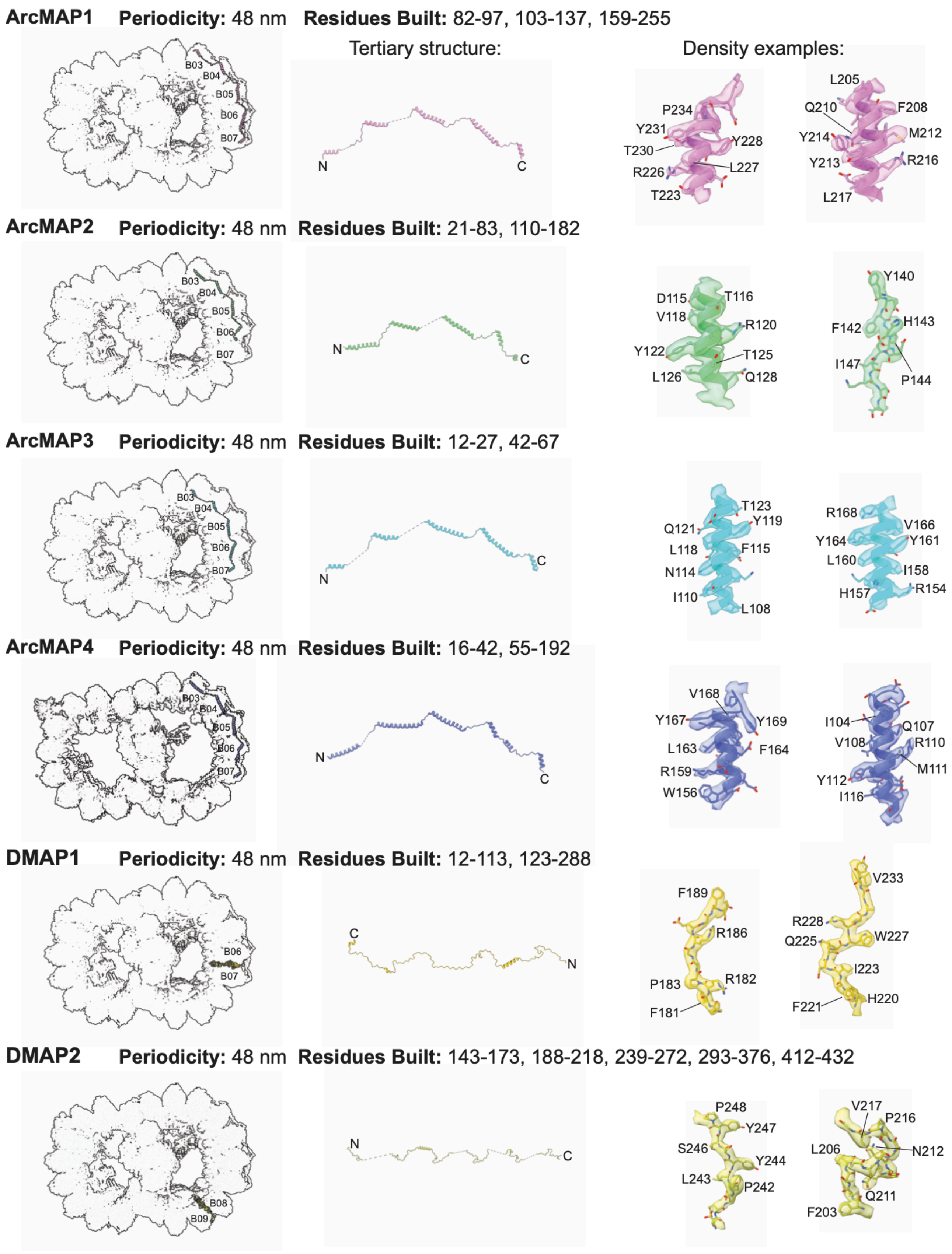

Figure S14. Location, tertiary structure, and density examples for doublet microtubule-associated proteins ArcMAP1-4, DMAP1 and DMAP2.

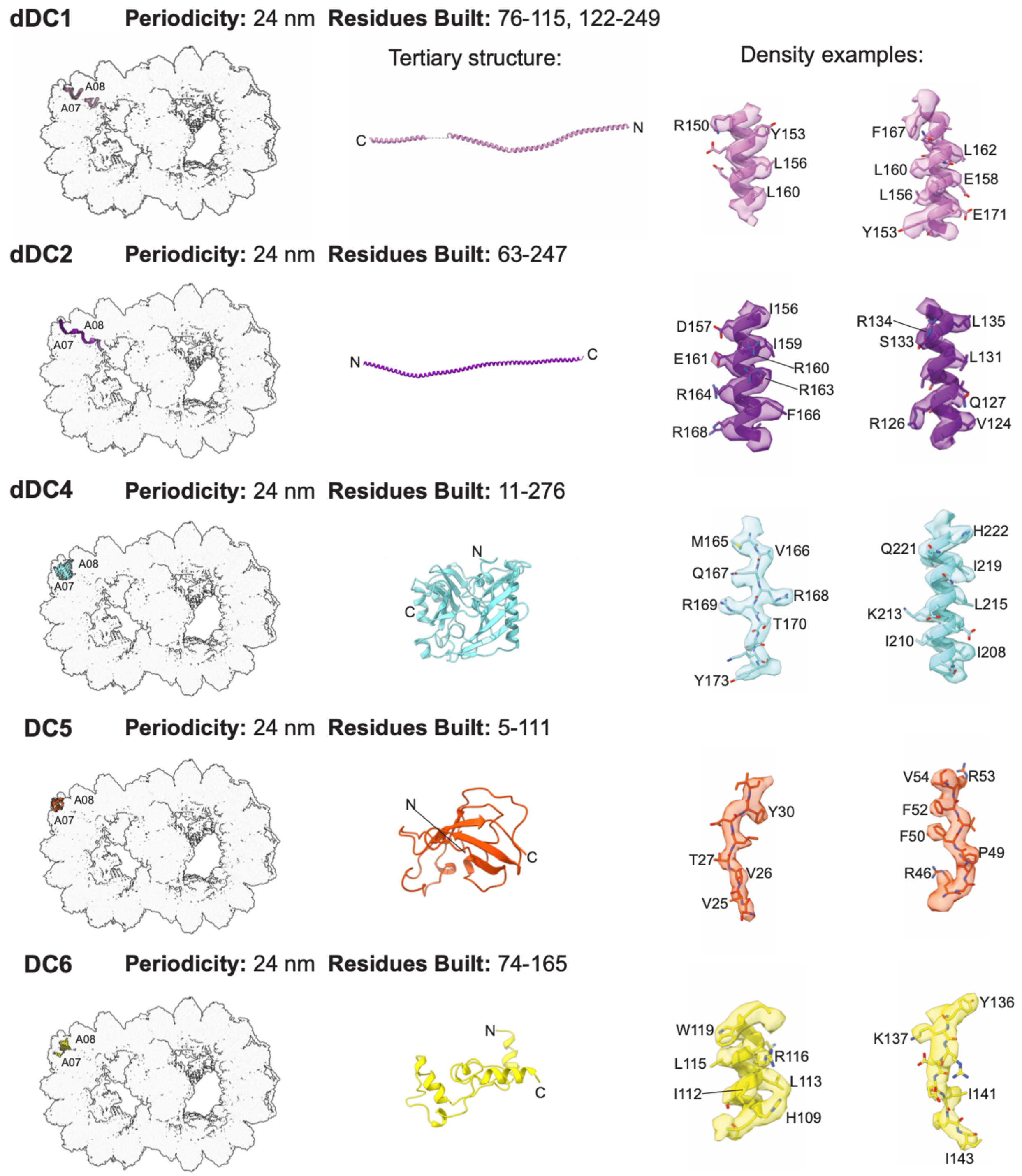

Figure S15. Location, tertiary structure, and density examples for the subunits of the distal outer dynein arm docking complex (ODA-DC).

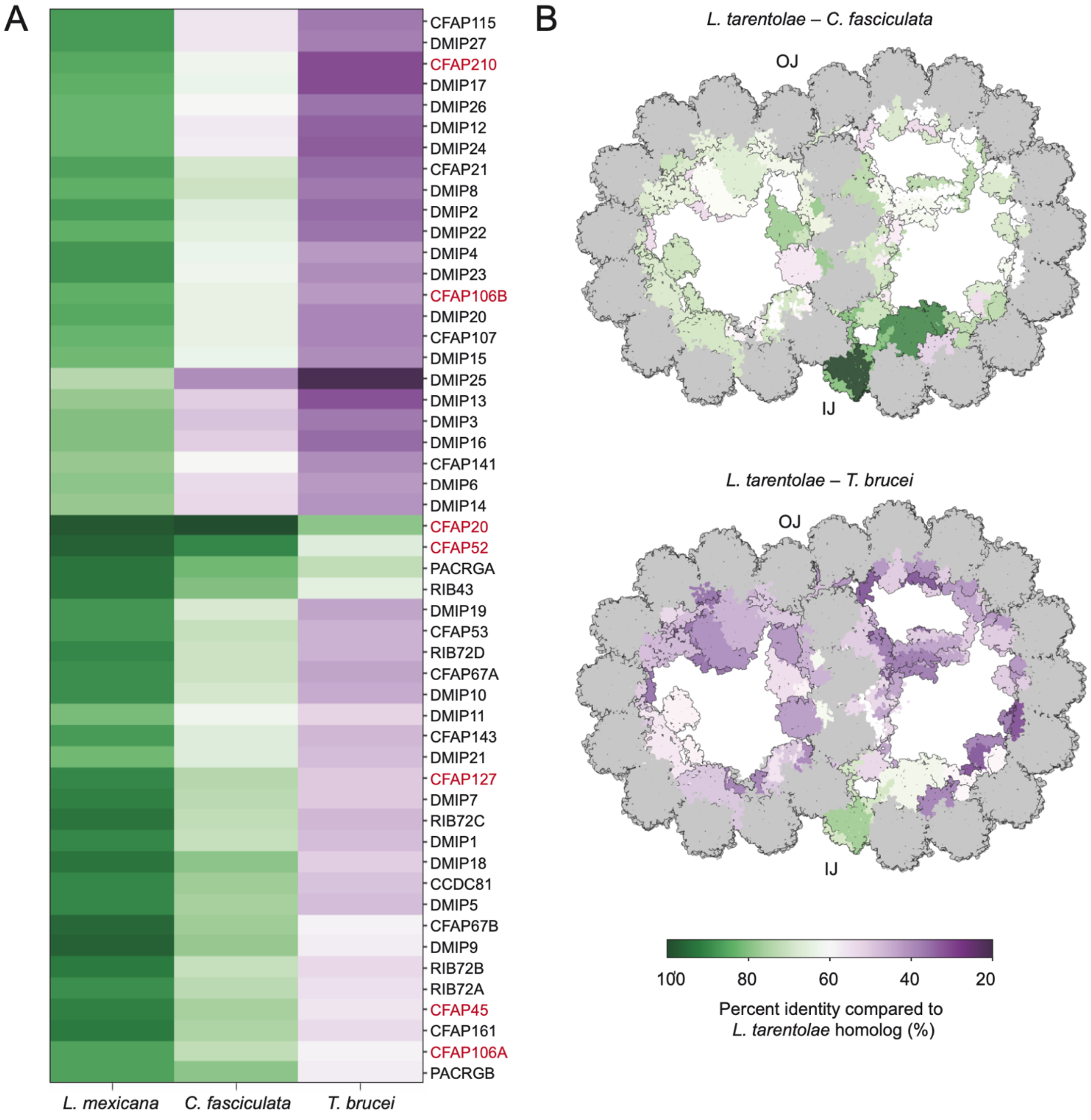

Figure S16. Conservation of trypanosomatid microtubule inner proteins (MIPs). (**A**) Heatmap comparing MIPs of *L. tarentolae* with three other species: *L. mexicana*, *C. fasciculata*, and *T. brucei*. Each cell represents the percentage of identical amino acid residues between a MIP in *L. tarentolae* and its corresponding homolog in the other species. MIPs were clustered using Ward's method of agglomerative hierarchical clustering. MIPs, in general, show high conservation among trypanosomatid species. For example, *L. tarentolae* MIPs share an average identity of 87% with *L. mexicana* homologs, 67% with *C. fasciculata* homologs, and 45% with *T. brucei* homologs. Core MIPs (labeled in red text) are among the most highly conserved MIPs across species. (**B**) Cross-sectional views of the *L. tarentolae* doublet microtubule with MIPs colored by percent sequence identity of homologous proteins in *C. fasciculata* (top) and *T. brucei* (bottom). Notably, proteins located at the inner junction (IJ) have the highest percent identity, suggesting a strong evolutionary conservation across species.

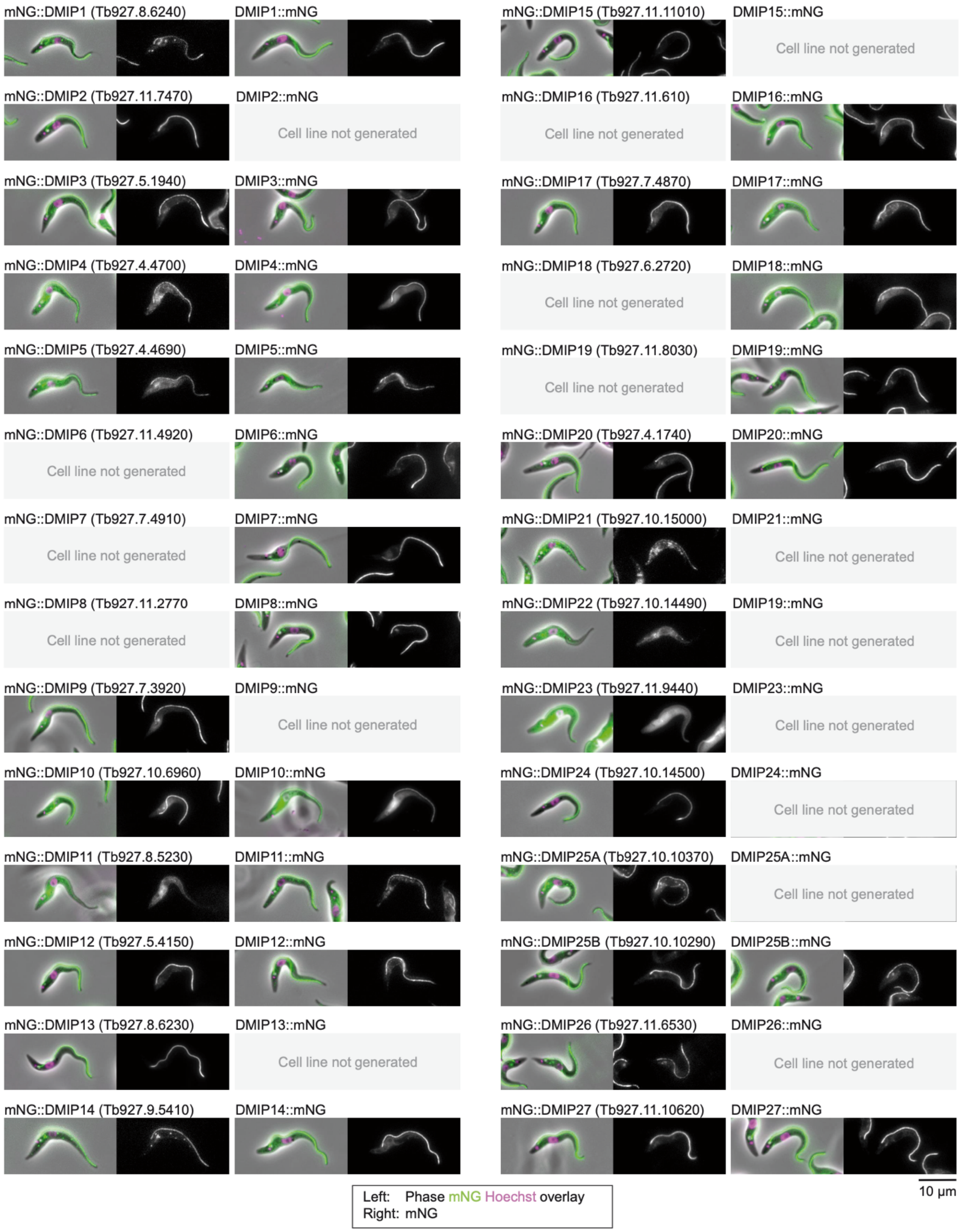

Figure S17. TrypTag localization data for doublet microtubule inner proteins (DMIPs). Examples of cells endogenously expressing DMIPs tagged with mNeonGreen (mNG) at either their N or C terminus, sourced from TrypTag. The left panel of each image displays an overlay of the mNG fluorescence signal (green), phase contrast (phase) and Hoechst 33342 (magenta), a fluorescent marker that stains nuclear and kinetoplast DNA. The right panel exclusively shows the isolated mNG signal. DMIP22 has a high cytoplasmic background and weak signal in the flagellum.

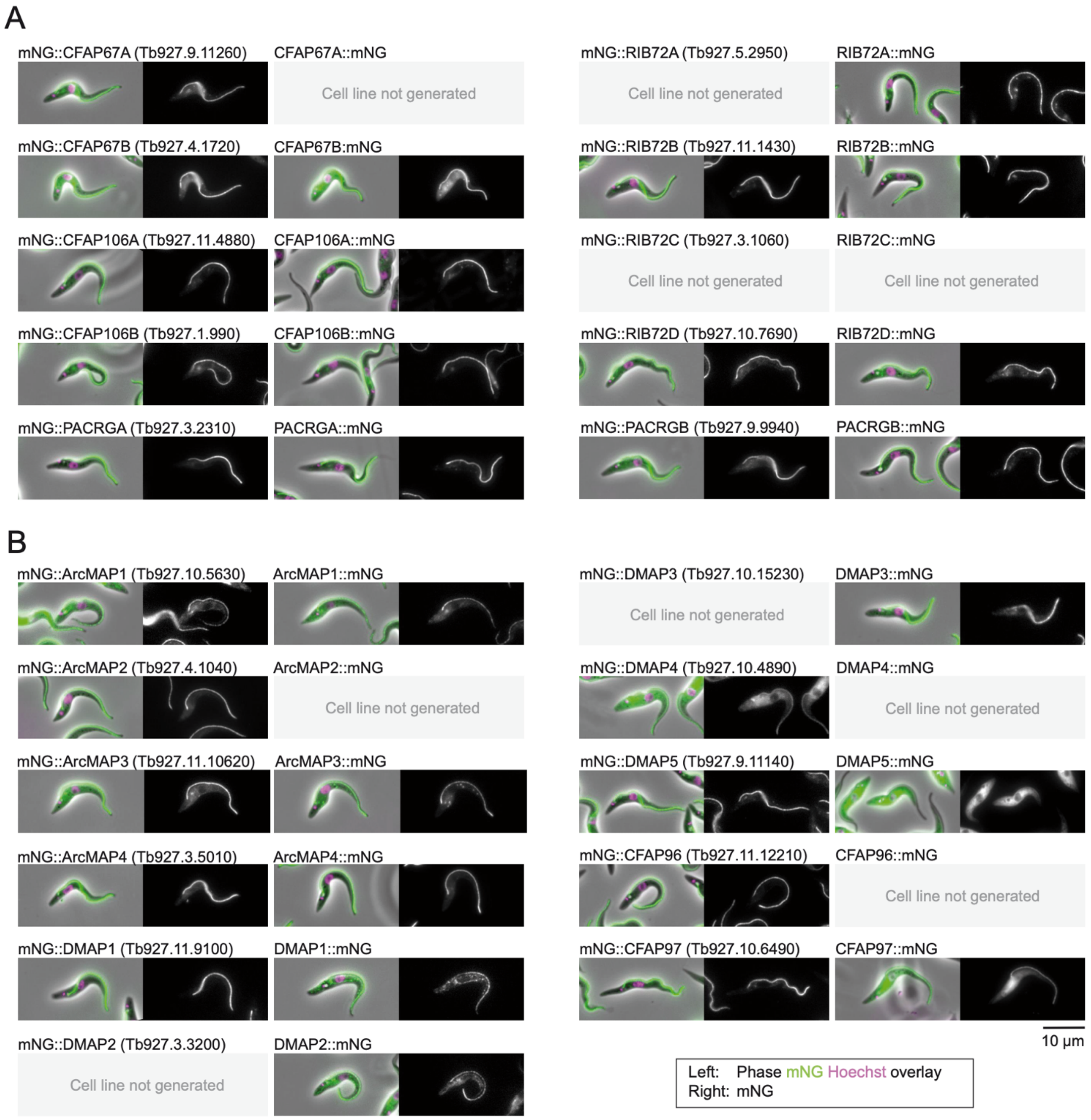

Figure S18. **TrypTag localization data for paralogous MIPs and external MAPs**. **A**. Examples of fluorescently tagged *T. brucei* cells showing that paralogous MIPs are expressed throughout the entire length of the flagellum and do not display proximal-distal asymmetry. Attempted tagging of RIB72C was unsuccessful. **B**. Examples of *T. brucei* cells expressing fluorescently tagged MAPs. The left panel of each image displays an overlay of a phase contrast image with fluorescence signal from mNeonGreen (mNG) and Hoechst 33342. The right panel shows just the mNG signal.

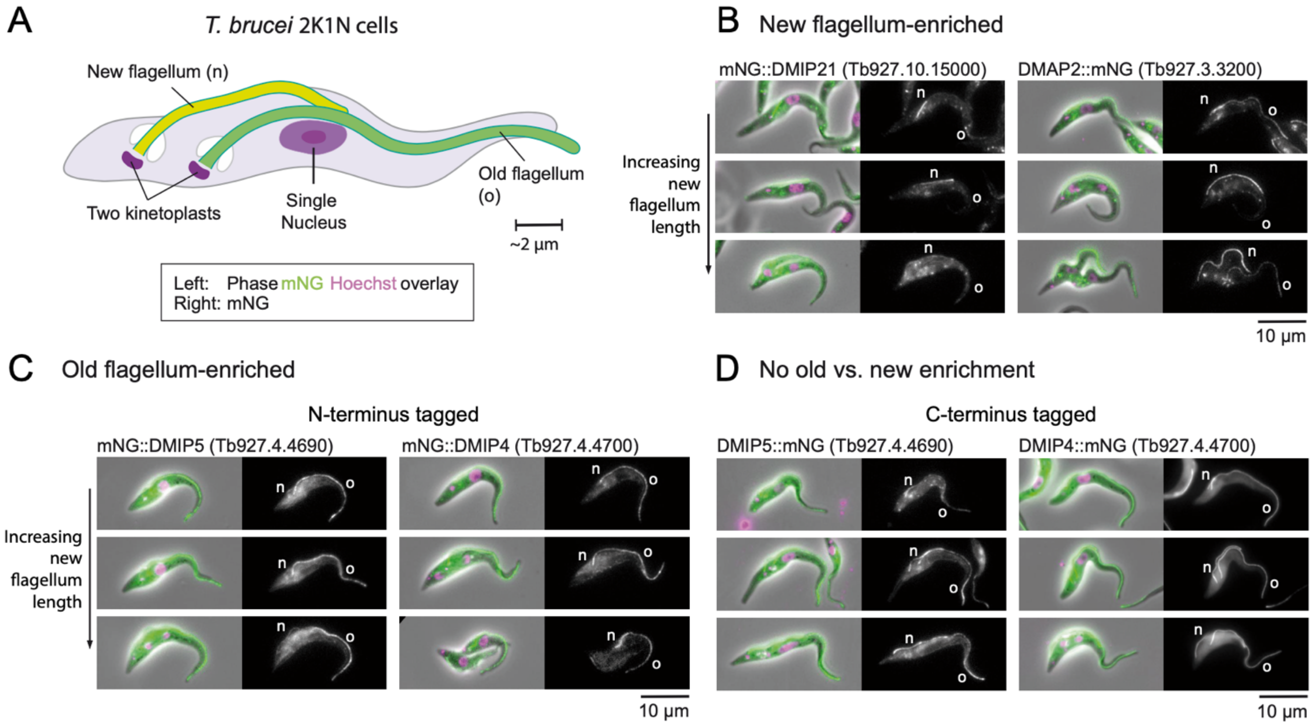

Figure S19. Proteins with cell-cycle-dependent localization to *T. brucei* flagella. (**A**) An illustration of a *T. brucei* 2K1N cell (with two kinetoplasts and one nucleus) that is actively assembling a new flagellum alongside the existing one. (**B**) TrypTag images of mNeonGreen (mNG) tagged DMIP21 and DMAP2 indicate a preference of these proteins for new flagella in 2K1N cells. (**C**) N-terminally tagged DMIP4 and DMIP5 are predominantly found in old flagella. (**D**) C-terminal tagging of DMIP4 and DMIP5 eliminates their preference for old flagella. All other *T. brucei* proteins examined do not display a preference for either new or old flagella.

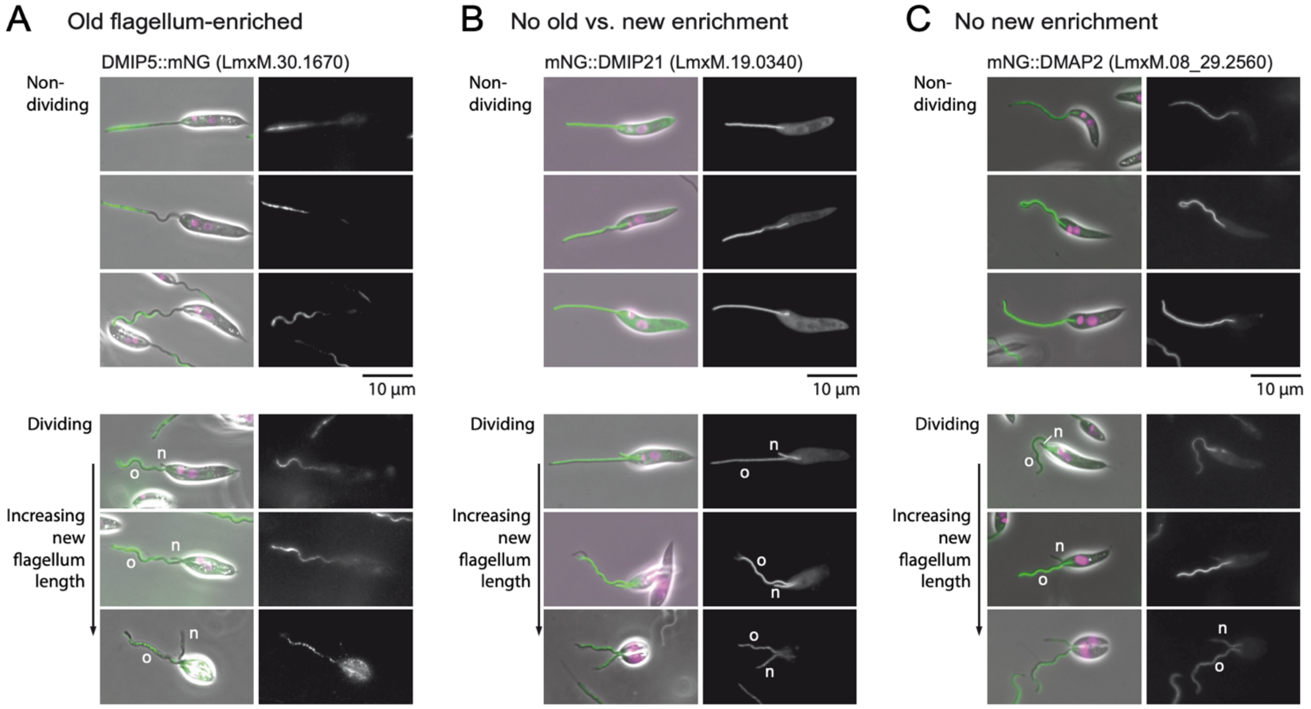

Figure S20. Tagging proteins with suspected cell-cycle-dependent localization in *L. mexicana*. (**A**) C-terminally tagged *L. mexicana* DMIP5 is predominantly found in the distal end of mature/long flagella. (**B**) N-terminally tagged *L. mexicana* DMIP21 shows no preference for old or new flagella. (**C**) N-terminally tagged *L. mexicana* DMAP2 is not enriched in new flagella. Indeed, signal in the new flagellum is weak and mostly confined to the proximal region, suggestive of slow incorporation, from base to tip, in the new flagellum.

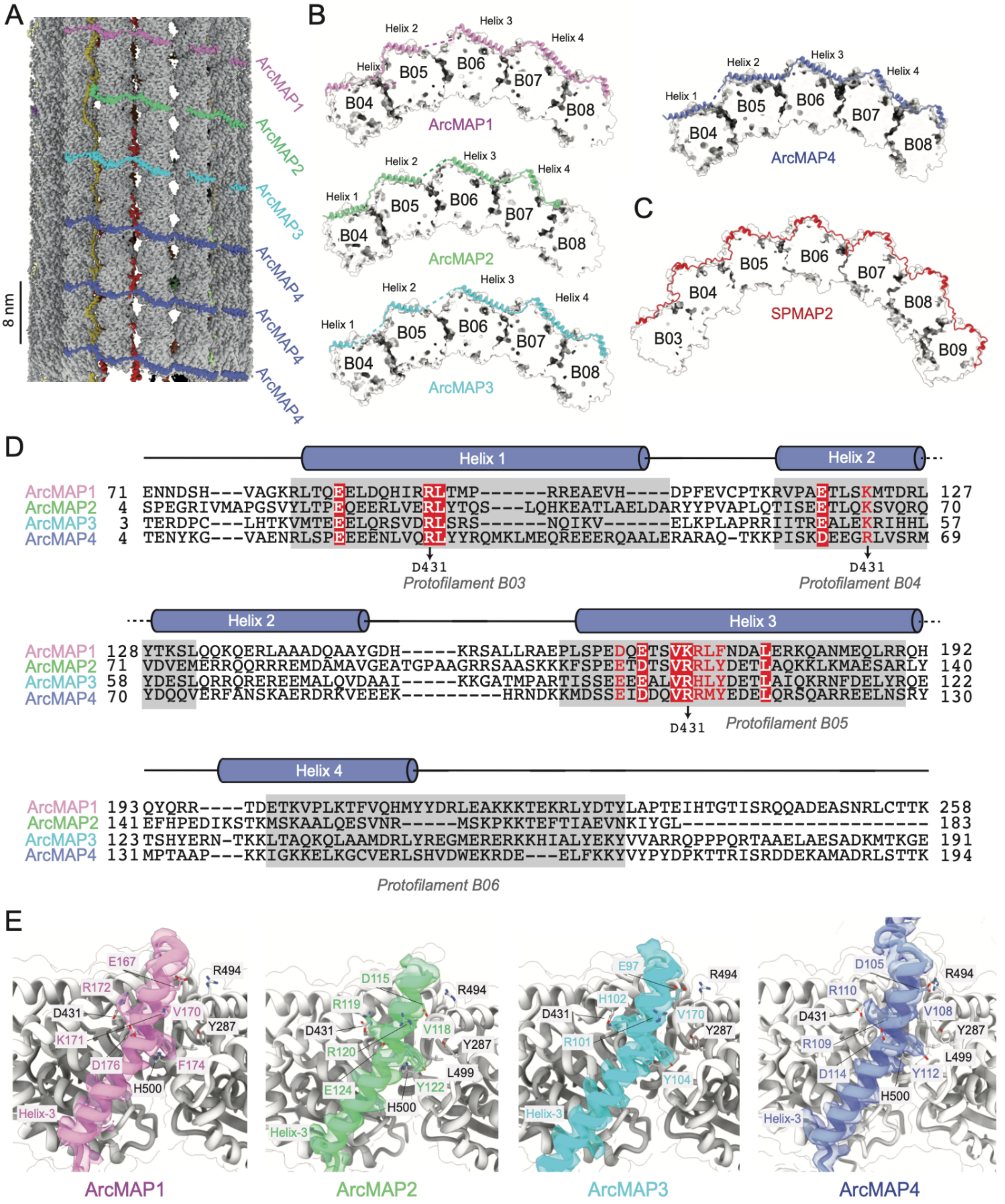

Figure S21. ArcMAP details. (**A**) Overview showing the positioning of ArcMAPs on the exterior of the *L. tarentolae* B tubule. (**B**) Cross sectional views of ArcMAPs 1-4 bound to B-tubule. (**C**) SPMAP2 occupies similar binding site in the bovine sperm doublet microtubule (PDB: 8OTZ). (**D**) Sequence alignment of the three ArcMAP paralogs. Residues with conserved properties are shown in red. Invariant residues are shown in white and highlighted in red. Secondary structure is shown above the sequences. Grey boxes represent the protofilaments to which the sequences are bound. (**E**) Details showing how helix 3 of the three ArcMAPs interacts with the tubulin exterior.

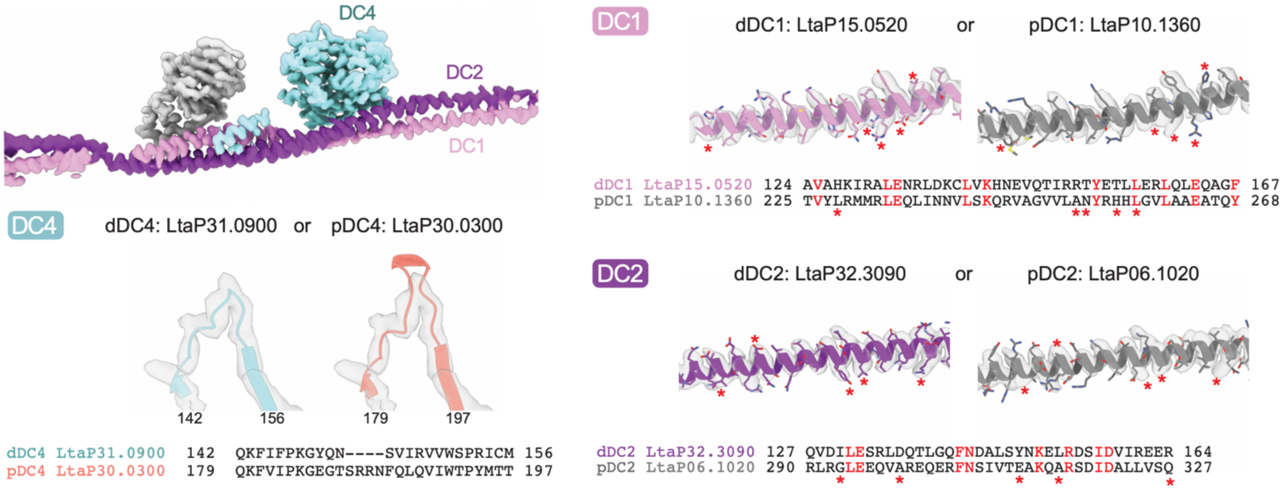

Figure S22. Identification of ODA-DC subunits. The *L. tarentolae* genome encodes two paralogs each of the ODA-DC subunits DC1, DC2 and DC4, whose gene products display proximal-distal asymmetries. Our cryo-EM density is consistent only with the distal paralogs. Differences in loop length (as demonstrated for DC4) or in individual sidechains (as demonstrated for DC1 and DC2) can distinguish these paralogs. Residues with notable differences between paralogs are marked with a red asterisk.

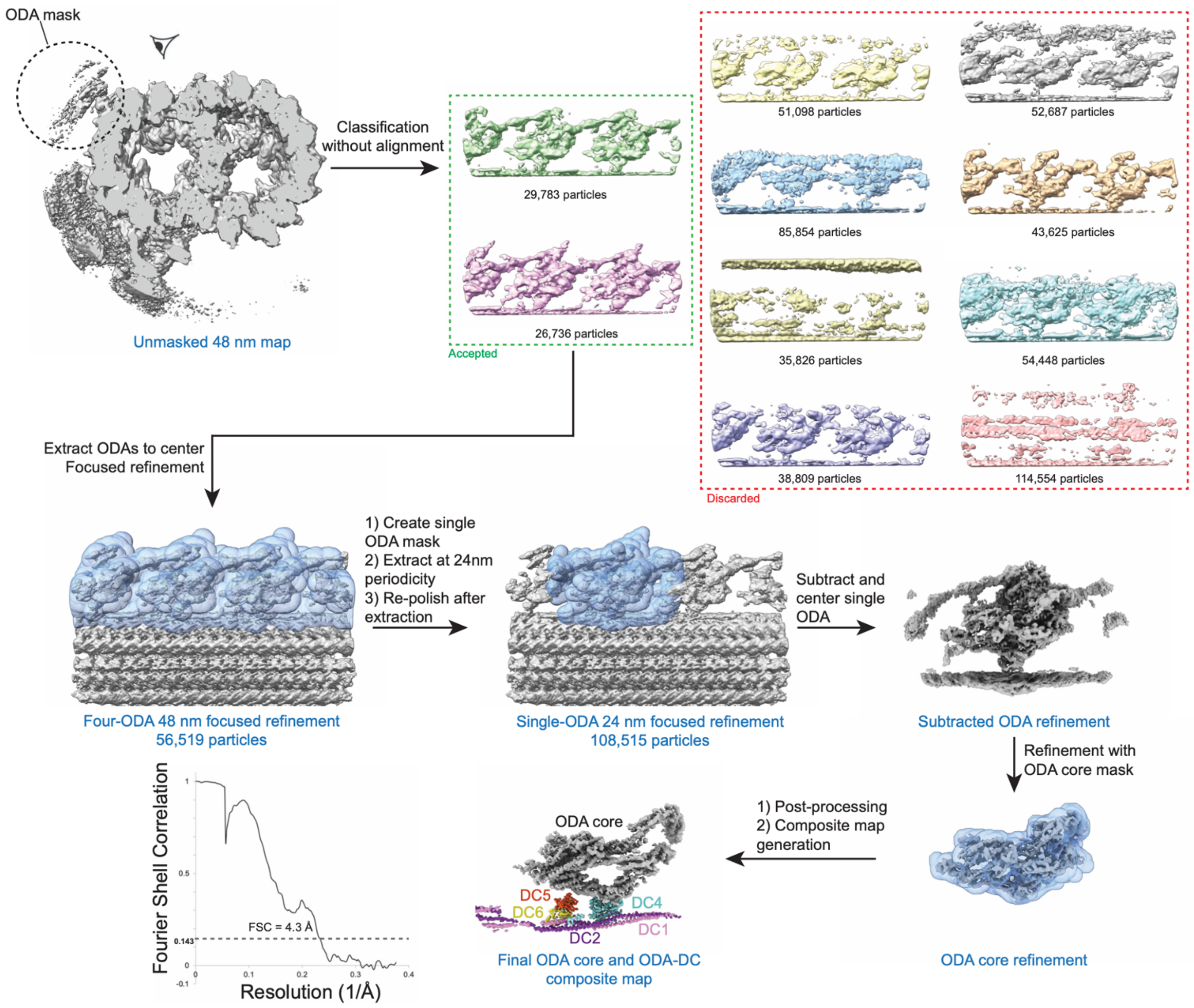

Figure S23. **Cryo-EM processing of the *L. tarentolae* outer dynein arm (ODA)**. Schematic of the processing pipeline used to improve the map of the ODA. Mask-focused classification without alignment was performed to isolate classes with robust ODA density (green box) starting with a consensus refinement of the 48-nm repeat of the doublet microtubule. Because ODAs repeat every 24 nm, these classes featured two ODAs. Each ODA was separately extracted and combined to generate a set of 108,515 particles used as input for mask-focused refinement. The map was combined with the composite map from fig. S1.

TABLES

	L. tarentolae DMT, 48 nm repeat (EMDB-47661) (PDB 9E78)	*C. fasciculata* DMT, 48 nm repeat (EMDB-47684)
**Data collection and processing**		
Facility	Harvard Cryo-EM Center for Structural Biology	Pacific Northwest Center for Cryo-EM (PNCC)
Microscope	Titan Krios	Titan Krios
Energy Filter	BioQuantum K3, 25 eV slit width (Gatan)	BioQuantum K3, 20 eV slit width (Gatan)
Detector	K3 (Gatan)	K3 (Gatan)
Magnification	64,000×	81,000×
Voltage (kV)	300	300
Electron exposure (e–/Å^2)	61-63	~60
Defocus range (μm)	−0.5 to −3.0	−0.7 to −2.5
Pixel size (Å)	1.33	1.061 Å / 1.0655 Å
Symmetry imposed	C1	C1
Movie stacks	37,665	38,511
Initial particle images (no.)	7,883,514	970,134
Final particle images (no.)	533,420	241,951
Map resolution range (Å)<br>FSC threshold	2.9-3.3<br>0.143	2.7-3.1<br>0.143
**Refinement**		
Model resolution (Å)<br>FSC threshold	3.5<br>0.5	
Map sharpening *B* factor (Å^2)	20	
Model composition<br>Chains<br>Non-hydrogen atoms<br>Protein residues<br>Ligands	 490<br>1,371,765<br>173,744<br>161	
B factors (Å^2)<br>Protein<br>Ligand	 85.54<br>77.37	
R.m.s. deviations<br>Bond lengths (Å)<br>Bond angles (°)	 0.003<br>0.688	
Validation<br>MolProbity score<br>Clashscore<br>Poor rotamers (%)	 1.93<br>16.61<br>0.05	
Ramachandran plot<br>Favored (%)<br>Allowed (%)<br>Disallowed (%)	 96.72<br>3.28<br>0.01	

Table S1. **Cryo-EM data collection, refinement and validation statistics**

Gene	Tagging terminus	Oligo sequence (5' to 3')
DMIP4	N	F: CTACCGACAGCATCATATACACCTCCGCCCgtgtatcggatgtcagttgcCCAGATCAAAAGCAATCgtataatgcagacctgctgc<br>R: TCCGTGCTGGCAGGTGGGGGCTGAGGGCATactacccgatcctgatccag<br>sg: gaaattaatacgactcactataggCTCCTGCTTGTACTATAGCGgttttagagctagaaatagc
DMIP4	C	F: AAGATGGCGGAGGCCATGCAGCGCAAAAAGggttctggtagtggttccgg<br>R: GAAGACTCATTGCACAGACCTGCTGAGCCAccaatttgagagacctgtgc<br>sg: gaaattaatacgactcactataggCATGGTGTTTTGTTCTGCTGgttttagagctagaaatagc
DMIP5	N	F: CGCATCACTGCACACTGACCGCACAAACCAgtgtatcggatgtcagttgcTGCGGTCCGCATCTTCAgtataatgcagacctgctgc<br>R: CGAAGGCAGCGTACCGGCGGTGATGCTCATactacccgatcctgatccag<br>sg: gaaattaatacgactcactataggAAGAGTTGTGAAGAGAGTGGgttttagagctagaaatagc
DMIP5	C	F: CGTGAGTACTATGCTGCCCAGGACCAGGAGggttctggtagtggttccgg<br>R: CTTTTCTGACATCTGGTCACCCTTGGGCCGccaatttgagagacctgtgc<br>sg: gaaattaatacgactcactataggTGATGTAACGTTGCACCCCAgttttagagctagaaatagc
DMIP21	N	F: TCGGCACACACTGTAAACAGCACAGGAAAAgtgtatcggatgtcagttgcCCTCCTATCTGGGCATTgtataatgcagacctgctgc<br>R: CCGCTCCTTCTTGTATTCCCCACCGATCATactacccgatcctgatccag<br>sg: gaaattaatacgactcactataggAAAAATATTTCACAGCCGTGgttttagagctagaaatagc
DMIP21	C	F: GTCAAGTCTCTCTGTGTGGTGCCACCAGTCggttctggtagtggttccgg<br>R: GTCATCTGTCGTGGCCATAGGTCTCGGGAGccaatttgagagacctgtgc<br>sg: gaaattaatacgactcactataggGCCGCCGCATCGCACGACGAgttttagagctagaaatagc
DMAP2	N	F: TCGCACTCACTCTCTCACCCGCTTTCGCCGgtgtatcggatgtcagttgcTAGCGACTCCGTTCAAAgtataatgcagacctgctgc<br>R: GAGACCACCATAAACGCTTTTGTCTGTCATactacccgatcctgatccag<br>sg: gaaattaatacgactcactataggGATTCGAGAAAGGAGTCCGAgttttagagctagaaatagc
DMAP2	C	F: GTAGTATCCTCGTGCCTTCGCCGGTTCTACggttctggtagtggttccgg<br>R: ACAAACACACACTCACACGGAAAGCAGGGCccaatttgagagacctgtgc<br>sg: gaaattaatacgactcactataggTGCGTAGGGGAGACTTTGGTgttttagagctagaaatagc

Table S2. **Oligos used for gene tagging in *L. mexicana***. Each gene was independently tagged at the N and C terminus with mNeonGreen. Listed are the oligo sequences of the forward (F) and reverse (R) primers and the single-guide RNA (sg) for each experiment.

Data S1. Mass spectrometry analysis. *L. tarentolae* and *C. fasciculata* proteins identified by mass spectrometry in the samples used for cryo-EM. Provided as an Excel file.

Data S2. Proteins identified in the cryo-EM maps and their orthologs in *L. mexicana* and *T. brucei*. Provided as an Excel file.

Data S3. Knockout data. Quantitative analysis of swim speed and directionality of the *L. mexicana* mutants. Provided as an Excel file.
